# Competitive binding between DDX21 and SIRT7 enhances NAT10-mediated ac^4^C modification to promote colorectal cancer metastasis and angiogenesis– DDX21 promotes colorectal cancer metastasis

**DOI:** 10.1038/s41419-025-07656-3

**Published:** 2025-04-29

**Authors:** Angxi Song, Bowen Liu, Wenjing Li, Bingyuan Chen, Pengkun Gui, Hao Zhang, Can Zhu, Yixin Xu, Tao Jiang, Jun Song

**Affiliations:** 1 Department of General Surgery, The Affiliated Hospital of Xuzhou Medical University, Xuzhou, China; 2 Institute of Digestive Diseases, Xuzhou Medical University, Xuzhou, China; 3 Central Laboratory, The Affiliated Hospital of Xuzhou Medical University, Xuzhou, China; 4 Central Laboratory, Xuzhou NO.1 people’s hospital, Xuzhou, China

**Keywords:** Colon cancer, Cell invasion

## Abstract

DExD- box helicase 21 (DDX21) is overexpressed in colorectal cancer (CRC) and is positively correlated with poor prognosis and the malignant phenotype of CRC. Functional characterization indicated that DDX21 promotes CRC metastasis and angiogenesis both in vitro and in vivo. N-acetyltransferase 10 (NAT10) is a key regulator of the N4-acetylcytidine (ac^4^C) modification of mRNA, regulating the stabilization of mRNA via ac^4^C modification. Here, we identified that DDX21 competitive binding with sirtuin 7 (SIRT7), inducing the overexpression of NAT10. Furthermore, DDX21 upregulates NAT10 expression to enhance ac^4^C modification and the stability of ATAD2, SOX4 and SNX5 mRNAs, which mediate CRC metastasis and angiogenesis. Overall, the present study revealed a mechanism of DDX21/NAT10-mediated mRNA stability in CRC, laying the foundation for the use of DDX21 as a therapeutic target to overcome metastasis and angiogenesis in CRC.

**Figure:**
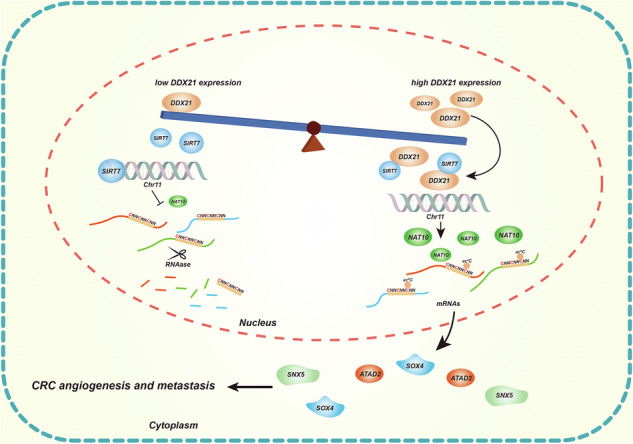
DDX21 competitive binding with sirtuin 7 (SIRT7), inducing the overexpression of NAT10. Furthermore, DDX21 upregulates NAT10 expression to enhance ac^4^C modification and the stability of ATAD2, SOX4 and SNX5 mRNAs, which mediate CRC metastasis and angiogenesis.

DDX21 competitive binding with sirtuin 7 (SIRT7), inducing the overexpression of NAT10. Furthermore, DDX21 upregulates NAT10 expression to enhance ac^4^C modification and the stability of ATAD2, SOX4 and SNX5 mRNAs, which mediate CRC metastasis and angiogenesis.

## Introduction

Colorectal cancer (CRC) is the third most common malignancy worldwide and the second leading cause of cancer-related mortality [1, 2]. Although the application of screening, neoadjuvant chemotherapy (NACT) and surgery has improved the therapeutic outcome of CRC patients in recent years, the prognosis of CRC patients remains poor [3–5]. Metastasis is the main cause of high mortality in CRC patients [6]. Angiogenesis results in the formation of a disordered and unstable vascular network that allows CRC cells to metastasize from the primary tumor site to distant organs [7]. Thus, angiogenesis is closely related to CRC metastasis, both of which are considered critical processes in the progression of CRC. Elucidating the mechanism of CRC metastasis and angiogenesis may provide promising strategies for CRC treatment.

DExD/H box proteins (DDX/DHX proteins) include a large number of proteins that are associated with several aspects of energy-dependent RNA metabolism, including translation, ribosome biogenesis, and alternative splicing [8]. Over the past few decades, studies have shown that the DDX/DHX proteins widely participate in tumor progression [9, 10]. DDX21, a DDX/DHX protein, is involved in tumor growth, DNA repair, and metastasis [11–13]. Previously, studies have indicated that DDX21 transcriptionally activates target genes. For instance, DDX21 induces the transcription of CEP55 and promotes neuroblastoma progression in a helicase-dependent manner [14]. In acute myeloid leukemia (AML), DDX21 recruits YBX1 to cooperatively trigger ULK1 expression and promote AML progression [15]. However, the role of DDX21 in CRC metastasis and angiogenesis remains unclear.

N4-acetylcytidine (ac^4^C) is a highly conserved modification of RNA [16] and is involved in the stability and translation of tRNA, rRNA, and mRNA. NAT10 is the only enzyme known to catalyze ac^4^C modification [17]. With the catalysis of NAT10, ac^4^C modification participates in tumor initiation, drug resistance, and development [17–19]. Despite accumulating evidence demonstrating the effect of ac^4^C modification on cancer development, the function of ac^4^C modification in CRC metastasis and angiogenesis remains unclear, and the relationship between DDX/DHX proteins and ac^4^C modification needs further exploration.

In the present study, we showed that DDX21 is upregulated in CRC. Upregulation of DDX21 is correlated with a malignant phenotype and poor prognosis in CRC patients. In addition, DDX21 drives CRC metastasis and angiogenesis both in vitro and in vivo. Mechanistically, DDX21 co-occupation the catalytic domain (CA domain) of sirtuin 7 (SIRT7), blocking SIRT7-mediated deacetylation of H3K18, transcriptionally activates NAT10. Furthermore, we found that the DDX21/NAT10 axis regulated ATAD2, SOX4 and SNX5 ac^4^C modification, upregulated the mRNAs stability and expression levels, and promoted CRC metastasis and angiogenesis. Overall, we showed that DDX21 contributes to CRC metastasis and angiogenesis. This study provides a molecular basis for the potential use of DDX21 as a target in CRC treatment.

## Methods

### Clinical specimens

CRC and corresponding adjacent normal in tissue microarray (TMA) were obtained from the Affiliated Hospital of Xuzhou Medical University (Xuzhou, China). The patient had not received chemoradiotherapy. Specific specimen-related information is documented in Additional file 1: Table S1. In addition, we collected fresh frozen tissues for qRT‒PCR analysis. The clinical information of patients were listed in Additional file 2: Table S2. The study follows the Declaration of Helsinki, and approved by the Ethics Committee of the Affiliated Hospital of Xuzhou Medical University (Xuzhou, China).

### Cell culture and cell treatment

The human CRC cell lines (HCT116 (Cat. No.: TCHu99), SW620 (Cat. No.: TCHu101), SW480 (Cat. No.: TCHu172), DLD-1 (Cat. No.: TCHu134), and LoVo(Cat. No.: TCHu82)) were sourced from the Cell Bank of the Chinese Academy of Science (Shanghai, China). The human normal colorectal epithelial cell line NCM460 (Cat. No.: CC4042) was sourced from the American Type Culture Collection (Virginia). Cells were cultured in DMEM (Keygen) medium containing 10% fetal bovine serum (FBS; ExCell Bio). Add 1% antibiotic or antifungal solution as appropriate. The cells were placed in an incubator (37 °C, 5% CO_2_). Specifically, DLD-1 cells were cultured in RPMI-1640 medium (Keygen).

### Cell transfection

Negative control shRNA (sh-NC) and DDX21 shRNA (sh-DDX21) were purchased from GenePharma (Shanghai, China). NAT10 and SIRT7 siRNAs were purchased from IBSBIO (Shanghai, China). The siRNA sequences are recorded in Additional file 3: Table S3. DDX21, NAT10, and SIRT7 were amplified from human cDNA as templates and cloned and inserted into the pcDNA3.1(+) vector (Invitrogen). The transfection reagent is jetPRIME® Polyplus Transfection (Polyplus-transfection) and all operations are performed according to the relevant instructions.

### RNA extraction, reverse transcription‒PCR and qRT‒PCR

Total RNA was isolated using TRIzol (Vazyme) following the relevant specification. mRNA was reverse transcribed into cDNA using the SweScript RT I First Strand cDNA Synthesis Kit (Servicebio) following the relevant specification. Relative quantification of DDX21, SIRT7 and NAT10 was conducted using the 2-ΔΔCT method; for tissue samples, 18S rRNA served as the internal control, while GAPDH was used for cell lines. Each reaction was performed independently in triplicate. ABI StepOne (Carlsbad) and 2 × SYBR Green qPCR Master Mix (High ROX) (Servicebio) was used for qPCR experiments. Associated primer sequence are listed in Additional file 3: Table S3.

### Transwell assays

This study employed a modified two-chamber culture system with Transwell inserts (Corning Incorporated), featuring an 8-micron pore size, to assess cell migration and invasion capabilities. Inserts were either coated with Matrigel (BD Biosciences) for invasion assays or left uncoated for migration assays. Transfected cells were seeded into the Transwell inserts, subsequently fixed using 4% paraformaldehyde solution, and stained with 1% crystal violet following incubation. Images of the cells were captured using an Olympus microscope. Image analysis was performed using ImageJ software to quantify the number of cells that had migrated or invaded through the pores. Each experimental condition was repeated three times to ensure reliability and consistency of the results.

### Wound healing assay

The cells in the incubation wells (1 × 10^6^) were wounded using a plastic pipette tip. After 24 h of incubation, the extent of wound closure was evaluated and documented by imaging. The area of cell migration within the demarcated dashed lines in the wells was quantified using ImageJ software (NIH) and normalized relative to the migration of control cells.

### Tube formation assay

After spreading, 100 µl of Matrigel™ (BD Biosciences) was added to each well of 48-well plates. The plates were then placed in a humidified incubator at 37 °C until the Matrigel solidified, typically taking ~30–40 min. Well-grown HUVECs (HMEC-1 cells) were suspended in the corresponding tumor-conditioned medium (TCM) to achieve a cell concentration of 2 × 10^5^ cells/ml. This cell suspension (200 µl per well) was added to the Matrigel-coated 48-well plate and cultured under standard conditions. Count under an optical microscope, the average number of tubes in 5 random fields was calculated for statistical analysis.

### Western blot, immunofluorescence (IF) and antibodies

RIPA lysis buffer (Vicmed) containing phenylmethylsulfonyl fluoride (PMSF) was added to the cell culture dish. After centrifugation at 14,000 rpm for 15 min at 4 °C, the lysates were clarified. The protein content in cell lysates was quantified, followed by separation using SDS‒polyacrylamide gel electrophoresis. Subsequently, proteins were transferred to a PVDF membrane, blocked with 5% skim milk (BD Biosciences) in PBST. Incubate overnight at 4 °C for primary antibody incubation. The next day, incubate with secondary antibody at room temperature for 2 h. Chemistar™ High-sig ECL western blotting Substrate (Tanon) was used to detect signals.

The experiment was conducted after reaching 80% cell confluence. The cells were fixed by adding 4% paraformaldehyde (PFA, Sigma Aldrich), permeabilized with 0.2% Triton X-100. Incubation with the primary antibody at 4 °C for 8 h, the samples were incubated at room temperature for 2 h with the secondary antibody. CoraLite488-conjugated goat anti-rabbit IgG (H + L) and CoraLite594-conjugated goat anti-rabbit IgG (H + L) were purchased from Proteintech (Wuhan, China). The cell nuclei were stained with DAPI reagent. Finally, statistical analysis is carried out.

Information about antibodies: DDX21 (1:1000, Proteintech), NAT10 (1:2000, ABclonal), SIRT7 (1:100, Santa Cruz), H3K18ac (1:5000, ABclonal), histone H3 (1:5000, Proteintech), and β-Tubulin (1:5000, Proteintech).

### RNA immunoprecipitation (RIP)

The cells were suspended in 1.5 ml of nucleus isolation buffer (containing 1.28 M sucrose, 40 mM Tris-HCl pH 7.5, 20 mM MgCl2, and 4% Triton X-100) along with 4 ml of Diethylpyrocarbonate water, and incubate on ice for 20 min. Following this, RIP buffer (0.5 mM DTT, 150 mM KCl, 5 mM EDTA, 0.5% NP40, 100 U/ml RNase inhibitor (ABclonal), 25 mM Tris (pH 7.4), and cocktail (Vicmed)) was added, and sonication was used to disrupt the cell nuclei. After centrifugation, retain the supernatant. Incubate overnight at 4 °C with antibody. Magnetic beads were introduced and incubated for 2 h at 4 °C, Retain the magnetic beads, add TRIzol for RNA extraction. Finally, the RNA was quantified using qRT-PCR.

### Chromatin immunoprecipitation (ChIP)

The cells were in good condition, after washing twice with PBS, they were cross-linked with formaldehyde for 10 min and then incubated with ChIP lysis buffer (50 mM Tris-HCl (pH 8.0), 150 mM NaCl, 0.1% deoxycholate, 5 mM EDTA, 1% Triton X-100, 150 mM NaCl, 100:1 RNase inhibitor and cocktail), incubate on ice for 20 min. Next, sonication was used to disrupt the cell nuclei. Incubate overnight at 4 °C with the corresponding antibodies. Subsequently, add magnetic beads and incubate at 4 °C for 2 h. The samples underwent Chelex-100 treatment and thermal lysis to extract DNA. Proteinase K was added, and the mixture was shaken at 1300 rpm at 55 °C. Retain supernatant after centrifugation, and the results were analyzed by qPCR assay.

### RNA pull-down assay

PBS was added to the cell culture dishes in good condition for cleaning, 1 mL of RIPA buffer containing cocktail and ribonuclease inhibitor was added, incubate on ice for 20 min. Ultrasound treatment is performed, retain supernatant after centrifugation. The supernatant incubated with the corresponding probes for 4 h, and then incubate with magnetic beads for 2 h. Finally, WB experiment was performed to observe the results. In vitro biotin-labeled mRNAs were transcribed with Biotin RNA Labeling Mix (Roche Corporation) and T7 RNA polymerase (APExBIO), treated with an RNase inhibitor, and purified with a clean-up kit (Promega Corporation). The biotinylated mRNAs were reconstituted in binding and washing buffer, then incubation with streptavidin agarose resin (Beyotime Biotechnology).

### Hematoxylin-eosin (HE) and immunohistochemistry (IHC) assays

Embedding cancer and adjacent tissues in paraffin, then cutting into 4-micrometer-thick sections. For HE staining, the tissue sections were stained with hematoxylin for 3 min followed by eosin for 5 s after dewaxing. For IHC, antigen retrieval was performed by incubating the tissue sections in boiling 0.01 mol/L citric acid buffer for 15 min. The sections were incubated with primary antibodies at 4 °C for overnight. After washing, the sections were incubated with a secondary antibody for 2 h. DAB solution (Dako Denmark A/S) and hematoxylin were then applied for staining, followed by sealing of the slides.

### AcRIP-sequencing (acRIP-seq)

AcRIP-seq was performed at Guangzhou Epibiotek (Guangzhou, China). The cell lysate was incubated with the corresponding antibody at 4 °C for 8 h, and then with protein A/G magnetic beads for another 2 h to obtain the precipitated RNA fragments. The ac^4^C-enriched RNA was purified with a Zymo RNA Clean and Concentrator-25 Kit (Zymo Research). The library was prepared with an EpiTM mini long RNA-seq kit (Epibiotek). Both the input samples (without immunoprecipitation) and the ac^4^C immunoprecipitated (IP) samples underwent 150-bp paired-end sequencing using an Illumina NovaSeq 6000 sequencer.

### RNA ac^4^C dot blot (DB)

Total RNA was extracted from cells using TRIzol (Vazyme) reagent, and RNA solution was then spotted onto a Hybond-N+ membrane (GE Health). UV cross-linking was performed for 20 min. The membrane was blocked with 5% skim milk (BD Biosciences) for 2 h, followed by incubation with corresponding antibodies for 2 h. The membrane was then exposed to a secondary antibody, which was conjugated with horseradish peroxidase and targeted against rabbit IgG. The membrane was subsequently subjected to visualization using Chemistar™ High-sig ECL western blotting Substrate (Tanon) to examine the experimental results.

### In vivo experiments

All animal-related studies were conducted under approval from the Institutional Animal Care and Use Committee (IACUC) of Xuzhou Medical University (Xuzhou, China). Four-week-old female BALB/c nude mice were purchased from Vital River (Beijing, China) and housed in a specific pathogen-free (SPF) animal facility. The facility provided appropriate lighting conditions and standard access to food and water. For the lung metastasis experiment, mice were divided into two groups (*n*  = 10 per group) and injected via the tail vein with cells: one group received HCT116 cells (2 × 10^6^) transfected with sh-DDX21, while the control group received corresponding control cells. After 7 weeks, PET-CT was used to observe the size of the metastases, and then the mice were killed, and lungs were dissected for observation or further experiments.

For the liver metastasis experiment, the grouping is the same as before. Cells were injected into the distal spleen following laparotomy. Mice received appropriate antibiotics or analgesics daily. After 6 weeks, PET-CT was used to observe the size of the metastasis, and then the mice were killed, and liver tissues were dissected for observation or further experimentation.

For the subcutaneous tumor model, the grouping is the same as before. HCT116 cells transfected with sh-DDX21 were injected into the left axilla of nude mice, while corresponding control cells were injected into the right axilla of the same mice (2 × 10^6^). Tumor size was observed and recorded daily. After 5 weeks, the mice were euthanized, and tumor models were dissected for observation or further experimentation. The tumor cannot exceed 2000 mm^3^ in mice.

Name of Analgesic drug: Flunixin meglumine, mode of administration: IM, dosage: 1.1 mg/kg, which can maintain antipyretic and analgesic effects for about 12 h. At the same time, roxithromycin ointment was applied to the surgical incision to prevent infection. At the same time, to prevent infection, roxithromycin ointment was applied to the surgical incision, which was then covered and wrapped with a dressing. The wound was checked daily, and the dressing was changed in a timely manner.

### PET/CT imaging studies

PET/CT scans were performed using a Micro-PET/CT system. Mice were injected via the tail vein with ~3.7 MBq (100 μCi) of 18F-FDG (fluorodeoxyglucose) and underwent continuous static PET scanning 30 min after injection. For biodistribution studies, mice were injected with 0.37 MBq (10 μCi) of 18F-FDG. One hour post-injection, the mice were euthanized and dissected, and the tumors were collected and weighed. Radioactivity was measured using a γ-counter, and the results are expressed as the percentage of injected dose per gram of tissue (%ID/g).

### Analysis of online datasets

The normalized DDX/DHX protein expression values in the online datasets were downloaded from the GEPIA dataset (http://gepia.cancer-pku.cn/). The correlation assays were performed on the TIMER2.0 online database (http://timer.cistrome.org/).

### Statistical analysis

Statistical analysis of all experimental data was performed using GraphPad Prism 8.0.2 software (San Diego, CA, USA). Each experiment was independently repeated three times to ensure reliability. Student’s *t*-test was employed to compare differences between two groups. One-way ANOVA was employed to evaluate differences among multiple groups. For categorical variables, either the chi-square test or Fisher’s exact test was applied as appropriate. Overall survival (OS) analysis was performed using the Kaplan-Meier method, with statistical significance defined as a two-tailed p-value less than 0.05.

## Results

### DDX21 is overexpressed in CRC and correlated with poor outcomes

**Fig. 1:**
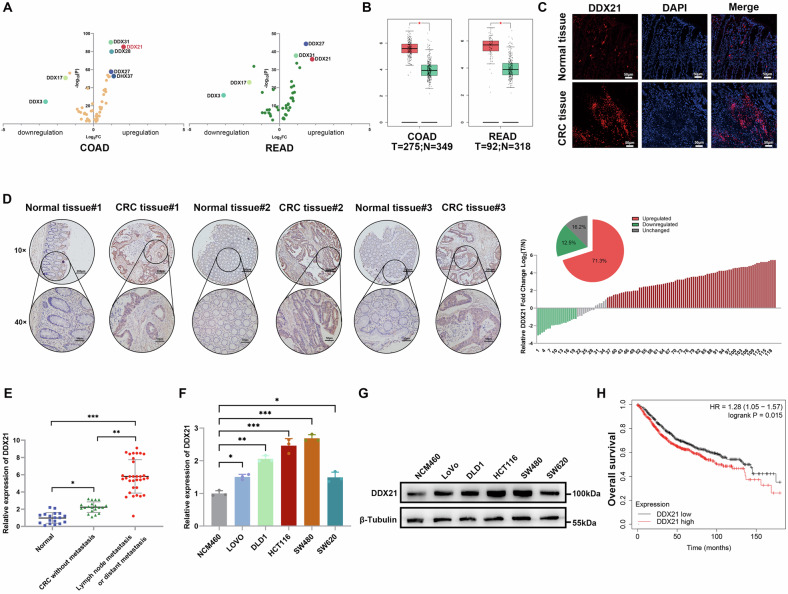
DDX21 was overexpressed in CRC and correlated with poor outcomes in CRC patients. **A** The differential expression levels of RNA helicases in CRC were identified via the TCGA database. **B** DDX21 expression levels in COAD (*T* = 275; *N* = 349) and READ (*T* = 92; *N* = 318) tissues were identified in the GEPIA database. **C** Representative images of IF staining for DDX21 in CRC tissues. DDX21 was labeled with CoraLite@594 (red), and nuclei were stained with DAPI (blue). Scale bars: 50 μm. **D** Representative images of IHC staining of DDX21 in CRC TMAs. The histogram and fan chart were used to show the statistical analysis of the IHC results. **E** qRT‒PCR was used to detect DDX21 expression in CRC tissues. **F**, **G** qRT‒PCR and western blotting were used to detect the expression levels of DDX21 in CRC cells and NCM460. **H** Kaplan‒Meier plot of overall survival via the Kaplan‒Meier plotter online database. Data are presented as mean ± SD. *P < 0.05, **P < 0.01, ***P < 0.001, compared with the corresponding control group.

First, the expression of DDX/DHX proteins was analyzed in TCGA database, and DDX21 exhibited the most significant difference in its expression level in CRC (Fig. 1A), and the mRNA level of DDX21 was significantly upregulated in the GEPIA dataset (Fig. 1B). Next, IF was used to examine DDX21 expression in CRC tissues (Fig. 1C). These results indicated that DDX21 is a potential oncogene in CRC. Therefore, we focused on DDX21 for further study. In addition, to determine the clinical significance of DDX21 in CRC patients, we performed a chi-square test of TMAs. The IHC staining showed that DDX21 was significantly upregulated in CRC tissues compared with normal tissues (Fig. 1D), and we found that increased DDX21 expression was positively associated with the malignant phenotype of CRC, such as tumor diameter (*p* = 0.0112), depth of invasion (*p* < 0.0001), lymph node metastasis (*p* = 0.0064), distant metastasis (*p* = 0.0027), and TNM stage (*p* = 0.0140), suggesting that DDX21 plays an oncogenic role in driving the progression of CRC (Additional file 1: Table S1). To verify the DDX21 expression pattern in CRC tissues, qRT‒PCR was used to determine its expression in normal tissues, CRC without metastasis and CRC with metastasis, and the results revealed that DDX21 was significantly upregulated in CRC (Fig. 1E). Furthermore, CRC patients with metastasis (including distant and lymph node metastasis) exhibited increased DDX21 expression (Fig. 1E). Then, we evaluated the mRNA and protein levels of DDX21 in human colon epithelial cells (NCM460) and five different CRC cell lines (LoVo, DLD1, HCT116, SW480, and SW620). We found that the expression of DDX21 in CRC cell lines was noticeably higher than that in NCM460 cells (Fig. 1F, G). Next, the Kaplan‒Meier plotter online database (http://kmplot.com/analysis/) showed that DDX21 was associated with poor overall survival (OS) (Fig. 1H). Overall, these results indicated that DDX21 was upregulated in CRC and correlated with poor outcomes in CRC patients.

### DDX21 promotes CRC metastasis and angiogenesis in vivo

**Fig. 2:**
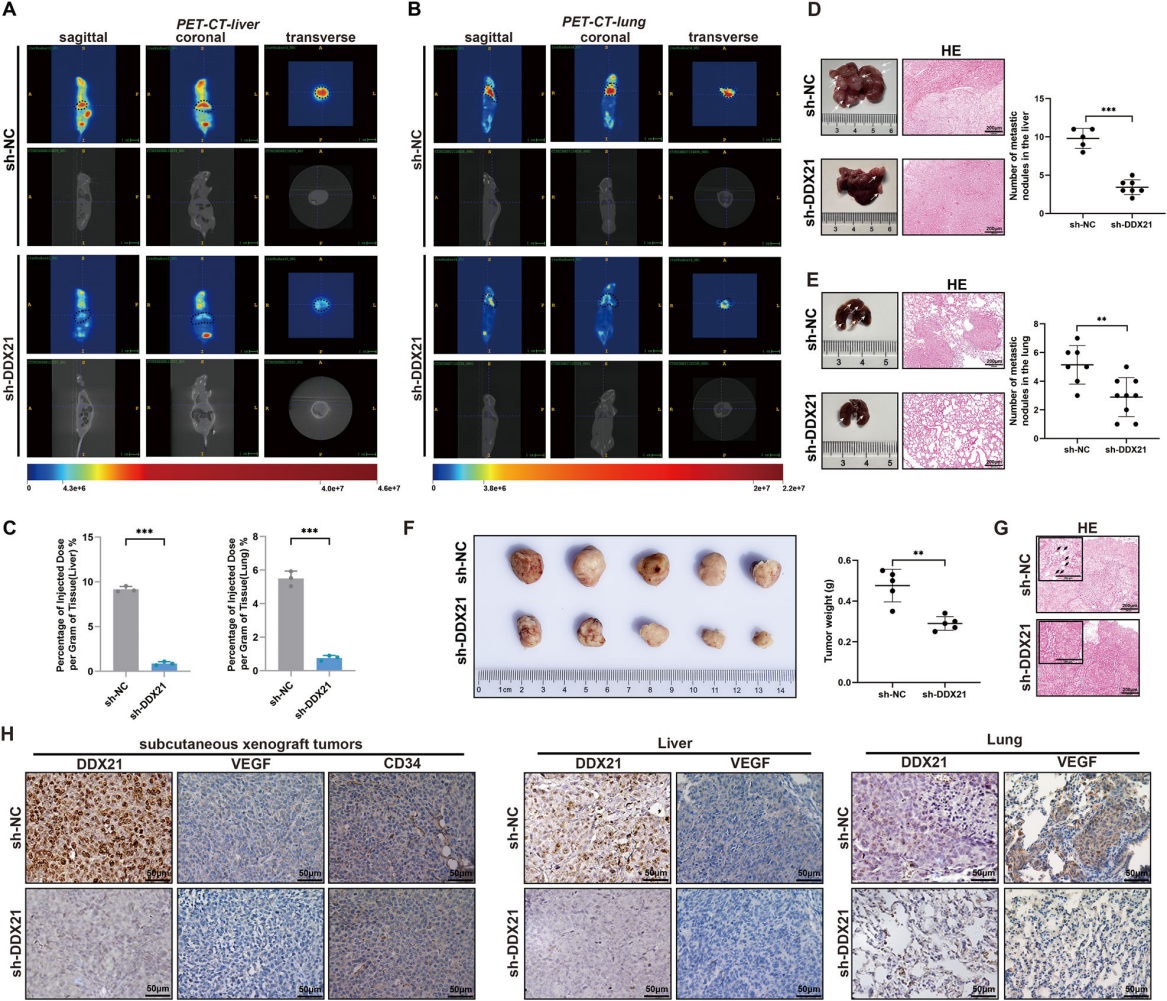
DDX21 promoted CRC metastasis and angiogenesis in vivo. **A**, **B** Representative PET‒CT images of lung (**A**) and liver (**B**) metastatic nodules and statistical analysis of SUV-related signal intensity. Scale bars: 1 cm. The signal intensity distribution in the livers or lungs of mice was measured using a γ-counter one hour after injection. **C** The results are expressed as the percentage of injected dose per gram of tissue (%ID/g). *n* = 3. Representative images of liver (**D**) and lung (**E**) metastatic. Statistical analysis of liver and lung metastasis foci. HE-stained liver and lung sections are shown. Scale bars: 200 μm. **F** Images and weight analysis of dissected subcutaneous xenograft tumors from different groups. **G** HE-staining showed vascular invasion in xenograft tumours. Scale bars: 200 μm. **H** IHC showed the expression level of DDX21 and VEGF in subcutaneous xenograft tumors and metastatic nodules, and the vascular invasion in subcutaneous xenograft tumors was observed using CD34. Scale bars: 50 μm. Data are presented as mean ± SD. *P < 0.05, **P < 0.01, ***P < 0.001, compared with the corresponding control group.

To address the biological significance of DDX21 in vivo, we silenced DDX21 in HCT116 cells with a DDX21-specific small hairpin RNA (sh-DDX21). We injected HCT116 cells with sh-DDX21 or negative control shRNA (sh-NC) into the tail vein or spleen of nude mice, PET-CT revealed more metastatic signals in the lungs or livers of mice injected with sh-NC than in those of mice injected with sh-DDX21 (Fig. 2A, B). The uptake of 18F-FDG in the lung or liver tissues of the sh-NC group mice was significantly higher than that in the sh-DDX21 group (Fig. 2C). Consistently, nude mice treated with sh-DDX21-transfected HCT116 cells generated fewer lung and liver metastatic nodules, while nude mice treated with sh-NC cells generated more lung and liver metastatic nodules than did their corresponding controls. HE staining revealed fewer metastatic foci in the livers and lungs of the sh-DDX21 group than the sh-NC group (Fig. 2D, E).

To further identify the role of DDX21 in CRC, we constructed subcutaneous xenograft tumors. The knockdown of DDX21 significantly inhibited tumor growth (Fig. 2F). In addition, we observed decreased vascular invasion in sh-DDX21-injected subcutaneous xenograft tumors (Fig. 2G). Next, IHC was used to detect the expression level of DDX21 and VEGF in sh-NC group and sh-DDX21 group, at the same time, vascular invasion was assessed by detecting CD34. The results showed that the sh-DDX21 group had lower VEGF expression levels in both subcutaneous xenograft tumors and metastatic nodules, as well as a lower vascular density (Fig. 2H). Taken together, these results revealed weaker metastasis ability and lower vascular invasion in the sh-DDX21 group and confirmed that DDX21-knockdown inhibited CRC metastasis and angiogenesis in vivo.

### DDX21 promotes CRC metastasis and angiogenesis in vitro

**Fig. 3:**
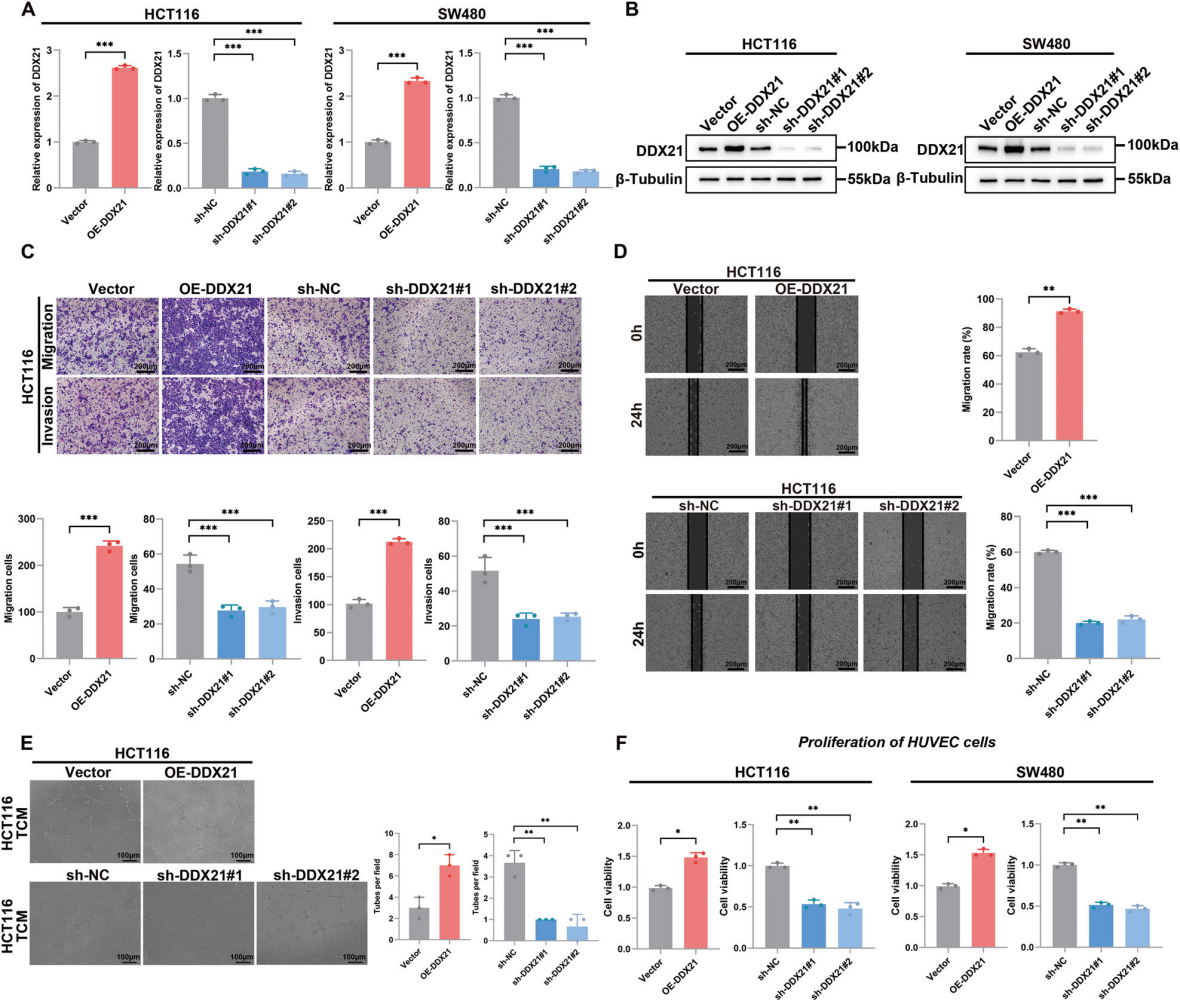
DDX21 promoted metastasis and angiogenesis in CRC. **A**, **B** qRT‒PCR and western blotting assays were used to detect the transfection efficiency. **C** The effects of DDX21 on cell invasion and migration were analyzed by Transwell assays. Scale bars: 200 μm. **D** The effects of different expression levels of DDX21 on cell migration were analyzed by wound healing experiments. Scale bars: 200 μm. **E** Tube formation assay was used to analyze the effects of DDX21 on angiogenesis in CRC cells. Scale bars: 100 μm. **F** DDX21 was knocked down or overexpressed in CRC cells, and the cells were cocultured with HUVECs. MTT assays were used to detect the proliferation of HUVECs. Data are presented as mean ± SD. *P < 0.05, **P < 0.01, ***P < 0.001, compared with the corresponding control group.

To investigate the underlying mechanism involved in DDX21-mediated metastasis and angiogenesis. We established DDX21-knockdown CRC cell lines in HCT116 and SW480 cells, and a DDX21 plasmid was subsequently constructed. The transfection efficiency in HCT116 and SW480 cells was measured by qRT‒PCR and western blotting (Fig. 3A, B). Then, Transwell (Fig. 3C, Additional file 4: Fig. S1A), wound healing (Fig. 3D, Additional file 1: Fig. S1B), and tube formation (Fig. 3E, Additional file 4: Fig. S1C) assays were performed using sh-DDX21 cells and DDX21-overexpressing cells. These results confirmed that DDX21 silencing significantly suppressed CRC metastasis and angiogenesis; in contrast, DDX21 overexpression enhanced the metastasis and angiogenesis abilities of CRC cells. To further confirm the function of DDX21 in enhancing angiogenesis, HUVECs were treated with the supernatant of CRC cells with DDX21 overexpression or knockdown. MTT assays revealed that DDX21 enhanced HUVEC proliferation (Fig. 3F). Next, ELISA confirmed that DDX21 promoted the expression of VEGF (Additional file 4: Fig. S1D) Previous studies have demonstrated that VEGF is a key regulator of angiogenesis [20, 21]. These results confirmed that DDX21 promoted CRC invasion, migration and angiogenesis, suggesting that DDX21 acts as a malignant regulator in CRC.

### DDX21 promotes metastasis and angiogenesis in CRC through NAT10

**Fig. 4:**
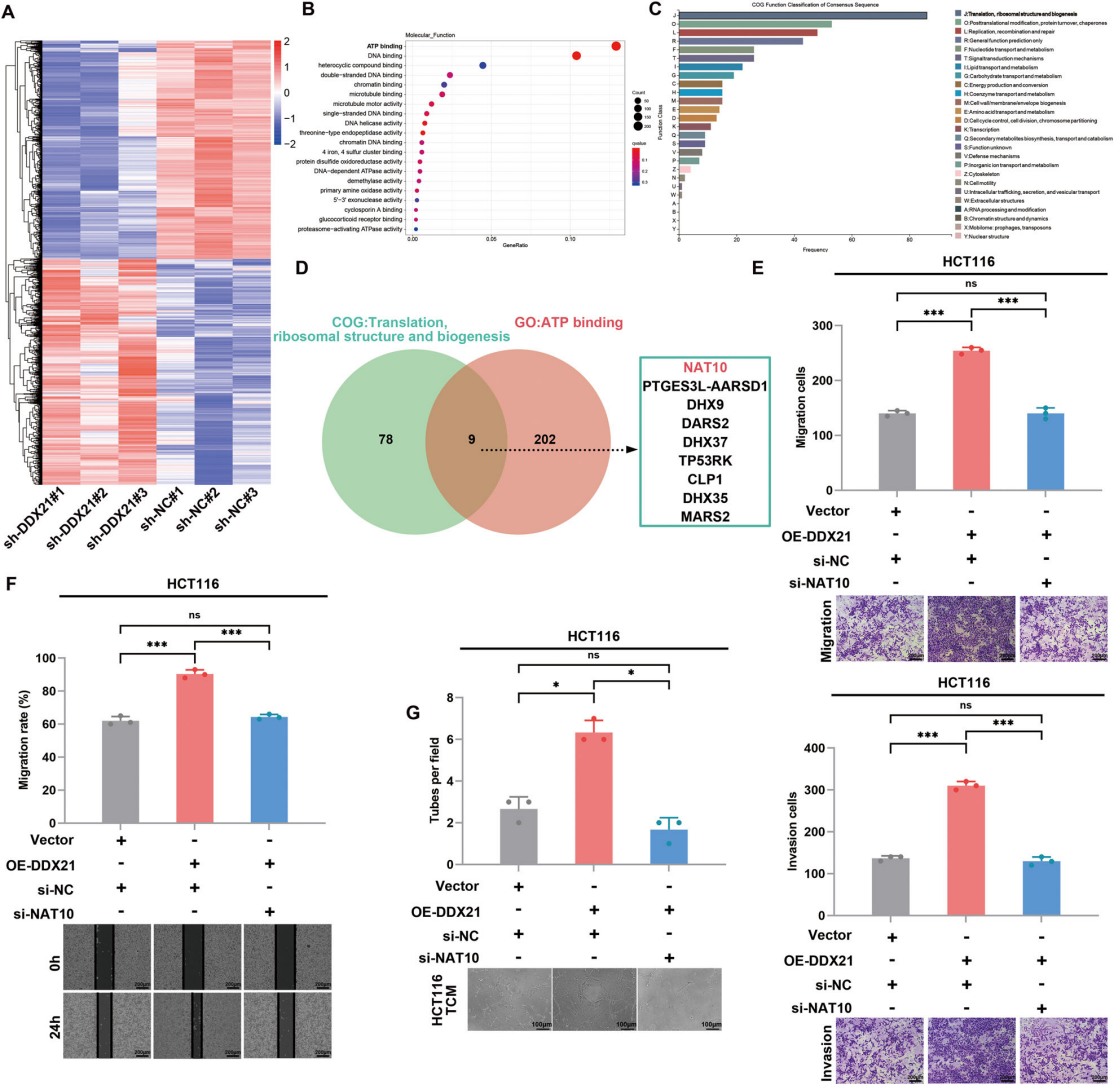
NAT10 is a target of DDX21 and promotes the metastasis and angiogenesis of CRC. **A** Gene expression patterns of DDX21-knockdown presented by heatmap. **B** GO analysis revealed the potential molecular function of DDX21. **C** COG functional classification of the DDX21 RNA-seq data. **D** Schematic drawing of the screening procedure for NAT10. **E** NAT10 was silenced in the DDX21-overexpressing cells, and the invasion and migration capacities of the cells were analyzed via Transwell assays. Scale bars: 200 μm. **F** NAT10 was silenced in the DDX21-overexpressing cells, and the ability of cell migration was analyzed by a wound healing assay. Scale bars: 200 μm. **G** NAT10 was silenced in the DDX21-overexpressing cells, and the ability of angiogenesis was analyzed by tube formation assay. Scale bars: 100 μm. Data are presented as mean ± SD. *P < 0.05, **P < 0.01, ***P < 0.001, compared with the corresponding control group.

To determine the function of DDX21 in CRC metastasis and angiogenesis, RNA sequencing (RNA-seq) was performed using HCT116 cells (Fig. 4A), and bioinformatics analysis was performed. Gene Ontology (GO) enrichment revealed that differentially expressed genes (DEGs) associated with DDX21 were enriched in ATP binding (Fig. 4B). In addition, all DEGs were subjected to Clusters of Orthologous Groups analysis, and the most overrepresented biological processes were involved in translation, ribosomal structure and biogenesis (Fig. 4C). The Venn diagram showed that 9 genes were enriched, with NAT10 being the most significantly different in the sh-DDX21 group (Fig. 4D). Next, qRT‒PCR and western blotting results demonstrated that the NAT10 expression level markedly decreased after DDX21 knockdown and increased after DDX21 overexpression (Additional file 4: Fig. S2A, B). These results demonstrated that DDX21 enhances NAT10 expression. Therefore, we identified NAT10 as a potential target of DDX21. To explore whether DDX21 promoted the metastasis and angiogenesis of CRC cells via a NAT10-mediated mechanism, rescue experiments were performed. The results showed that NAT10 knockdown reversed the effect of DDX21 on CRC cell migration, invasion and tube formation (Fig. 4E–G, Additional file 4: Fig. S2C–E). Taken together, these results demonstrated that DDX21 promoted CRC metastasis and angiogenesis in a NAT10-dependent manner.

**Fig. 5:**
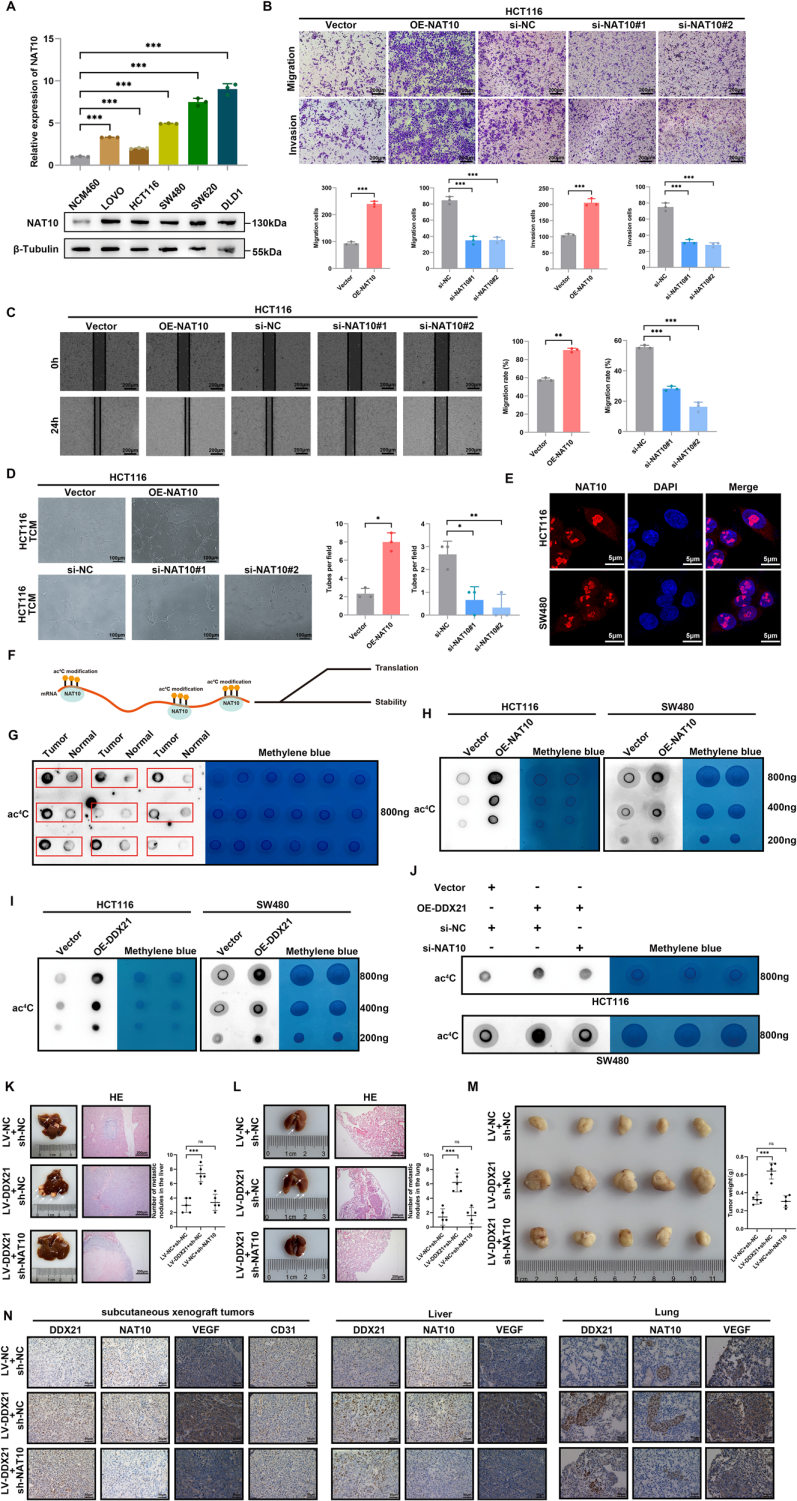
DDX21 promoted CRC metastasis and angiogenesis through NAT10-mediated ac^4^C modification. **A** qRT‒PCR and western blotting assays of NAT10 mRNA and protein expression in CRC cells. **B** The effects of NAT10 on cell invasion and migration were analyzed by Transwell assays. Scale bars: 200 μm. **C** The effects of different expression levels of NAT10 on cell migration were analyzed by wound healing experiments. Scale bars: 200 μm. **D** Tube formation assay was used to analyze the effects of NAT10 on angiogenesis in CRC cells. Scale bars: 100 μm. **E** Subcellular localization analysis of NAT10 in CRC cells via IF. NAT10 was labeled with CoraLite@594 (red), and nuclei were stained with DAPI (blue). Scale bars: 10 μm. **F** NAT10 mediates ac^4^C modification in RNA to promote RNA stability and translation. **G** Left: dot-blot assay of RNA ac^4^C modification in CRC patient samples. Right: semiquantification of the data shown on the left. **H**, **I** Dot-blot assay of RNA ac^4^C modification in CRC cells overexpressing DDX21 or NAT10. **J** Ac^4^C modification level of DDX21-overexpressing CRC cells transfected with NAT10 siRNA. Representative images of liver (**K**) and lung (**L**) metastatic. Statistical analysis of liver and lung metastasis foci. HE-stained liver and lung sections are shown. Scale bars: 200 μm. **M** Images and weight analysis of dissected subcutaneous xenograft tumors from different groups. **N** IHC showed the expression level of DDX21, NAT10 and VEGF in subcutaneous xenograft tumors and metastatic nodules, and the vascular density in subcutaneous xenograft tumors was observed using CD34. Scale bars: 50 μm. Data are presented as mean ± SD. *P < 0.05, **P < 0.01, ***P < 0.001, compared with the corresponding control group.

NAT10 is an acetylation enzyme that has been proven to participate in tumor progression. In addition, to investigate the role of NAT10 in CRC, qRT‒PCR and western blotting were used to explore the expression level of NAT10 in CRC cell lines and tissues (Fig. 5A, Additional file 4: Fig. S2F), and we found that NAT10 expression was significantly upregulated in CRC. We further investigated the effect of NAT10 on metastasis and angiogenesis using Transwell, wound healing and tube formation assays (Fig. 5B–D, Additional file 4: Fig. S2G–I). The results showed that NAT10 enhances CRC metastasis and angiogenesis.

Next, the results of IF assays confirmed the localization of NAT10, and NAT10 was found to be predominantly localized in the nucleus in dot-like structures (Fig. 5E). As the only known regulator of N4-acetylcytidine (ac^4^C) modification, NAT10 mediates ac^4^C modification in mRNAs, tRNAs and rRNAs, promoting RNA stability and translation (Fig. 5F). To investigate the role of mRNA ac^4^C modification in CRC, the total ac^4^C acetylation of RNA was quantified by Dot blot analysis, which showed that ac^4^C modification was significantly increased in CRC (Fig. 5G, Additional file 4: Fig. S2J). Next, NAT10 was overexpressed in HCT116 and SW480 cells, and the results of dot blot analysis showed that ac^4^C modification was significantly increased (Fig. 5H, Additional file 4: Fig. S2K). Interestingly, we observed increased ac^4^C modification with DDX21 overexpression (Fig. 5I, Additional file 4: Fig. S2L). Furthermore, dot blot analysis confirmed that NAT10 knockdown reversed DDX21-mediated ac^4^C modification (Fig. 5J, Additional file 4: Fig. S2M). These findings indicated that DDX21 enhances the level of ac^4^C modification in CRC via a NAT10-mediated mechanism. To further investigate the role of ac^4^C modification in DDX21-mediated metastasis and angiogenesis. We introduced Remodelin hydrobromide (REM) [22]. Next, we treated DDX21-overexpressing CRC cells with REM and performed Transwell, wound healing, and tube formation assays (Additional file 4: Fig. S3A–F). The results showed that REM can significantly reverse the promoting effects of DDX21 overexpression on CRC cell metastasis and angiogenesis. This finding clarified that DDX21-mediated CRC metastasis and angiogenesis depend on NAT10-mediated ac^4^C modification.

Furthermore, to verify the in vivo contribution of the DDX21/NAT10 axis to promoting CRC metastasis and angiogenesis, we performed metastasis models and subcutaneous xenograft models. Knockdown of NAT10 markedly inhibited DDX21 mediated cell growth and metastasis (Fig. 5K–M). Next, IHC revealed that DDX21 overexpression increased NAT10 expression, and increased vascular invasion (Fig. 5N).

### DDX21 enhances mRNA stability via NAT10-mediated ac^4^C modification

**Fig. 6:**
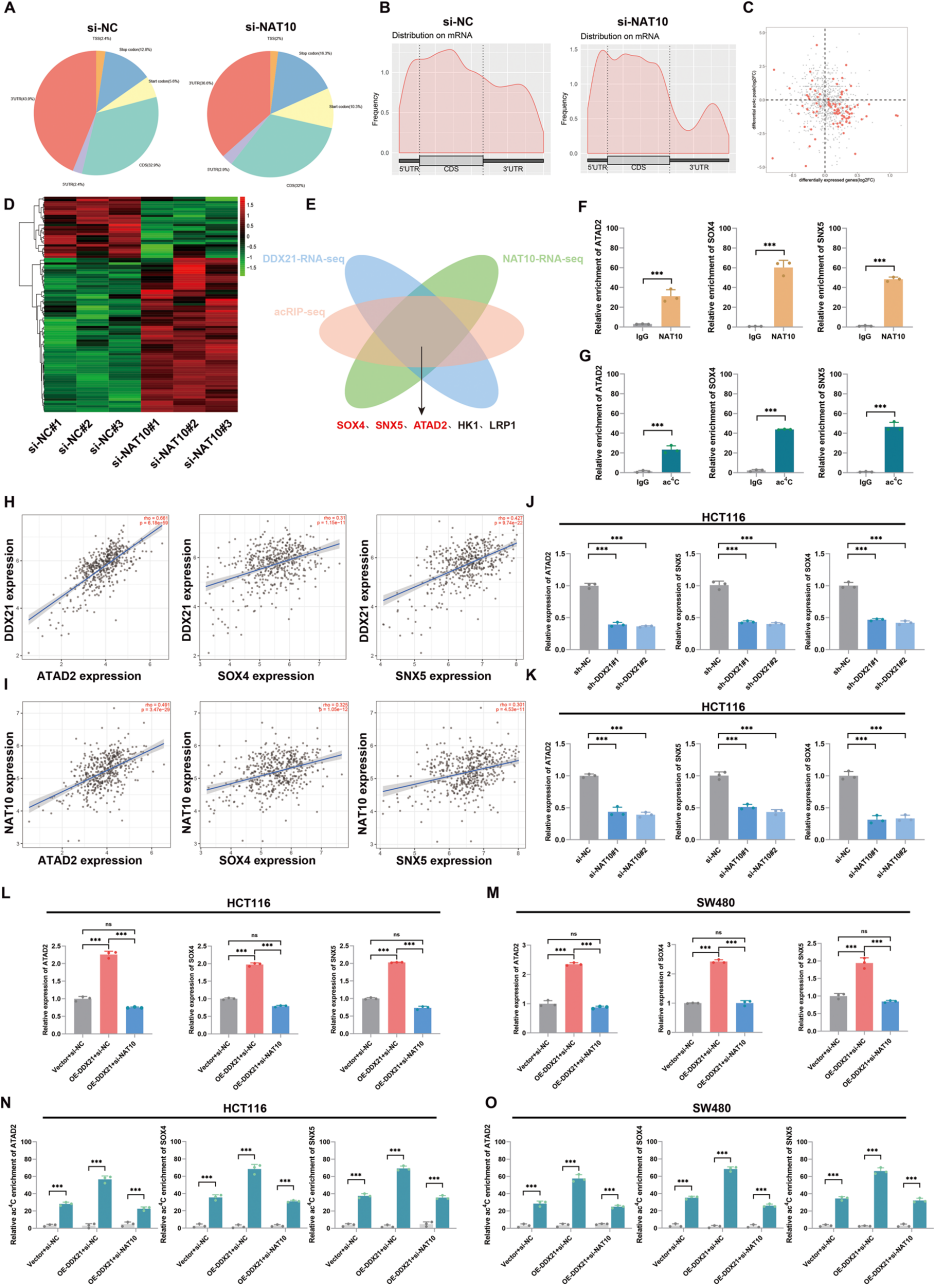
The DDX21/NAT10 axis remodeled SOX4, SNX5 and ATAD2 ac^4^C modification of mRNAs. **A**–**C** AcRIP-seq was performed to obtain the expression profiles of genes regulated by NAT10-mediated ac^4^C modification. A fan chart was used to show the main RNA ac^4^C modification enrichment sites in CRC (**A**). Based on the identified ac^4^C peaks, a metageplot plot was drawn, which reflects the distribution of peaks in the RNA structure (**B**). The four-quadrant diagram shows the correlation analysis between genes with changes in ac^4^C modification and genes with changes in RNA expression levels (**C**). **D** Gene expression patterns of NAT10-knockdown presented by heatmap. **E** Schematic drawing of the screening procedure or DDX21/NAT10 axis-regulated genes. **F**, **G** NAT10-RIP (**F**) and acRIP (**G**) were used to determine the enrichment of NAT10 or ac^4^C modification in mRNAs. **H**, **I** Correlation assays showing the relationships of DDX21 and NAT10 expression with ATAD2, SOX4, and SNX5 mRNA expression. **J** DDX21 knockdown inhibited the mRNA expression of ATAD2, SOX4, and SNX5 in HCT116 cells. **K** NAT10 knockdown inhibited the mRNA expression of ATAD2, SOX4, and SNX5 in HCT116 cells. **L**, **M** Relative expression of ATAD2, SOX4, and SNX5 mRNAs in DDX21-overexpressing CRC cells transfected with NAT10 siRNA, as assessed by qRT‒PCR. **N**, **O** Knockdown of NAT10 was performed in DDX21-overexpressing CRC cells, followed by acRIP-qPCR experiments to assess the levels of ac^4^C modification on ATAD2, SOX4, and SNX5. Data are presented as mean ± SD. *P < 0.05, **P < 0.01, ***P < 0.001, compared with the corresponding control group.

To obtain the expression profiles of genes regulated by NAT10-mediated ac^4^C modification, acetylated RNA immunoprecipitation sequencing (acRIP-seq) and NAT10-RNA-seq were performed (Fig. 6A–D). The results showed that ac^4^C modification was widely distributed across the transcriptome. Ac^4^C modification was enriched mainly in the 3’UTR and CDS regions of mRNAs, and NAT10 knockdown influenced mainly the CDS and 3’UTR modifications (Fig. 6B). To study the genes regulated by DDX21 and NAT10-mediated ac^4^C modification, DDX21-RNA-seq, NAT10-RNA-seq, and acRIP-seq were integrated. A Venn diagram indicated that five genes were downregulated with DDX21 or NAT10 silencing, and ac^4^C enrichment decreased with NAT10 knockdown (Fig. 6E). Among the five genes, SOX4, SNX5, and ATAD2 have been demonstrated to contribute to tumor metastasis and angiogenesis [23–27]. Moreover, ATAD2, SOX4 and SNX5 were more highly expressed in CRC tissues than in normal tissues; however, there was no significant difference in the expression levels of HK1 and LRP1 between CRC and normal tissues (Additional file 4: Fig. S4A–E). Then, we wondered whether DDX21-mediated ATAD2, SOX4, and SNX5 upregulation promotes CRC metastasis and angiogenesis via NAT10-mediated ac^4^C modification.

**Fig. 7:**
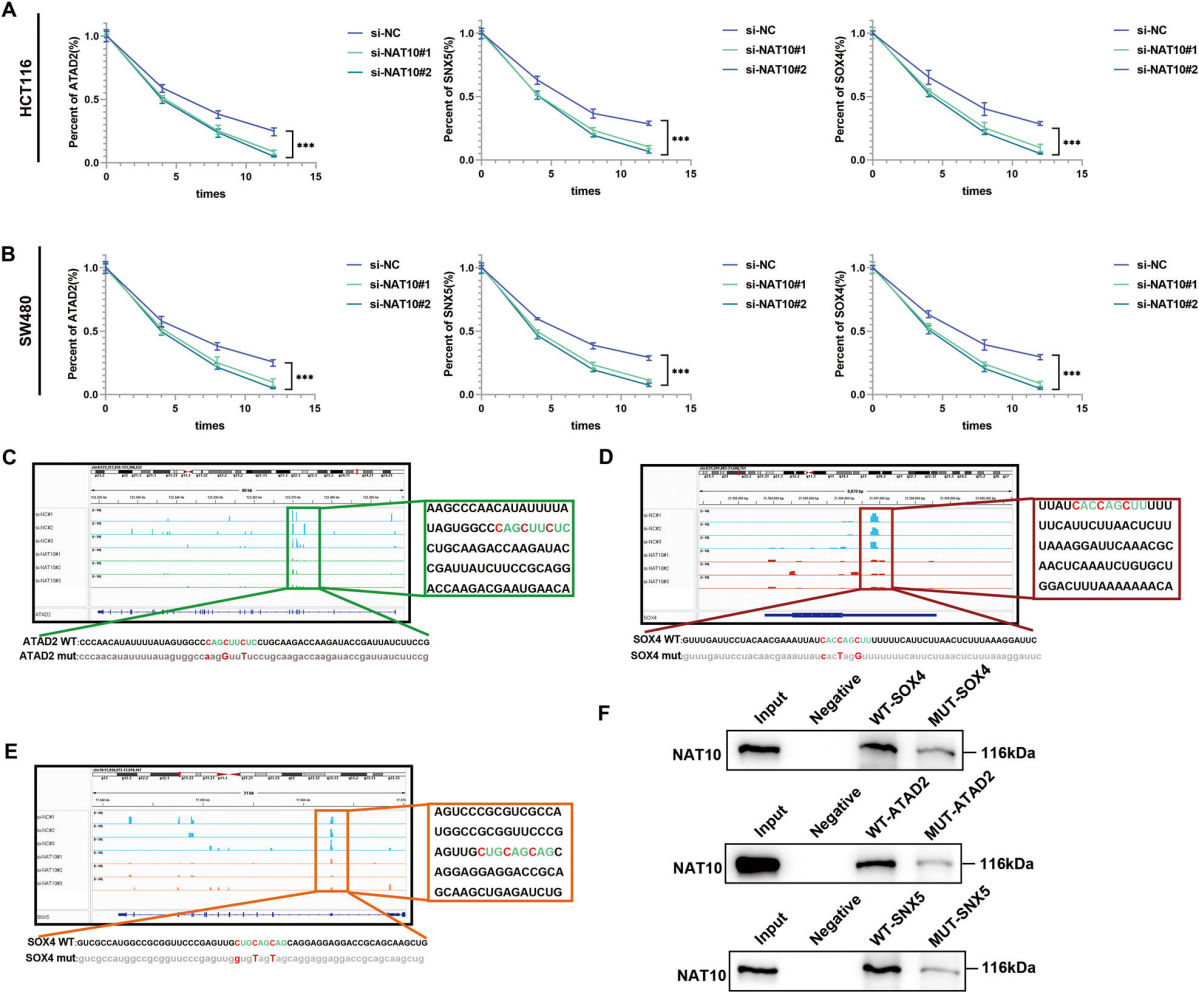
ATAD2, SOX4 and SNX5 were stabilized by NAT10-mediated ac^4^C modification. **A**, **B** An ACTD assay was used to detect mRNA stability with NAT10 silencing. **C**–**E** IGV was used to determine the ac^4^C modification peaks with the most significant changes after silencing NAT10, and ATAD2, SOX4, and SNX5 mutant plasmids were designed based on the data. **F** Western blotting of NAT10 in samples precipitated from biotinylated wild type or mutant mRNAs. Data are presented as mean ± SD. *P < 0.05, **P < 0.01, ***P < 0.001, compared with the corresponding control group.

To test our hypothesis, NAT10-RIP-qPCR and acRIP-qPCR were conducted, and marked NAT10 and ac^4^C enrichment was observed for the ATAD2, SOX4, and SNX5 mRNAs (Fig. 6F, G). RNA-seq indicated changes in the expression levels of these mRNAs, and ac^4^C modification should be dominated by mRNA stability in the regulation of these mRNAs. Next, the TIMER 2.0 database was used to predict correlations between genes. As expected, positive correlations were observed between the expression of DDX21 and NAT10 and that of ATAD2, SOX4, and SNX5 (Fig. 6H, I). The qRT‒PCR results demonstrated that ATAD2, SOX4, and SNX5 expression levels were significantly lower after DDX21 or NAT10 knockdown (Fig. 6J, K, Additional file 4: Fig. S4F, G). In addition, rescue experiments showed that the DDX21-mediated ATAD2, SOX4 and SNX5 upregulation was reversed by NAT10 knockdown (Fig. 6L, M). Next, we knocked down NAT10 in DDX21-overexpressing CRC cells and performed acRIP-qPCR experiments to assess the levels of ac^4^C modification on ATAD2, SOX4, and SNX5. The results showed that NAT10 knocked down reversed the DDX21-mediated ATAD2, SOX4, and SNX5 ac^4^C enrichment (Fig. 6N, O). We then overexpressed or silenced DDX21, and the loss of the mRNAs was measured after treatment with actinomycin D (ACTD) to block new RNA synthesis in HCT116 and SW480 cells. DDX21 overexpression enhanced ATAD2, SNX5 and SOX4 stability (Additional file 4: Fig. S5A), and silencing DDX21 significantly decreased ATAD2, SNX5 and SOX4 mRNA stability (Additional file 4: Fig. S5B). As expected, NAT10 knockdown decreased mRNA stability (Fig. 7A, B). A rescue test demonstrated that NAT10 knockdown inhibited DDX21 overexpression-mediated mRNAs stability (Additional file 4: Fig. S5C). The results revealed that DDX21 enhanced ATAD2, SOX4, and SNX5 mRNA stability via NAT10-mediated ac^4^C modification.

To elucidate the exact function of ac^4^C modification in CRC, Integrative Genomics View (IGV) software was used to search for potential ac^4^C modification sites based on our acRIP-seq datasets (Fig. 7C–E). Our acRIP-seq data revealed that ATAD2 and SNX5 mRNAs were modified by ac^4^C acetylation in the CDS region and that SOX4 mRNA was modified in the 3’UTR. Based on the ac^4^C peak diagram of genes from our acRIP-seq data, we selected the most common ac^4^C motif, “CXXCXXCXX”, with high confidence, and wild-type (WT) and mutant (mut) plasmids were designed (Fig. 7C–E). RNA pulldown assays revealed that the binding efficiency of the mutated mRNAs to NAT10 significantly decreased (Fig. 7F). Taken together, our data demonstrated that ATAD2, SOX4, and SNX5 are critical downstream targets of the DDX21/NAT10 axis.

### DDX21 promotes NAT10 transcription by competitively binding to the catalytic domain of SIRT7

**Fig. 8:**
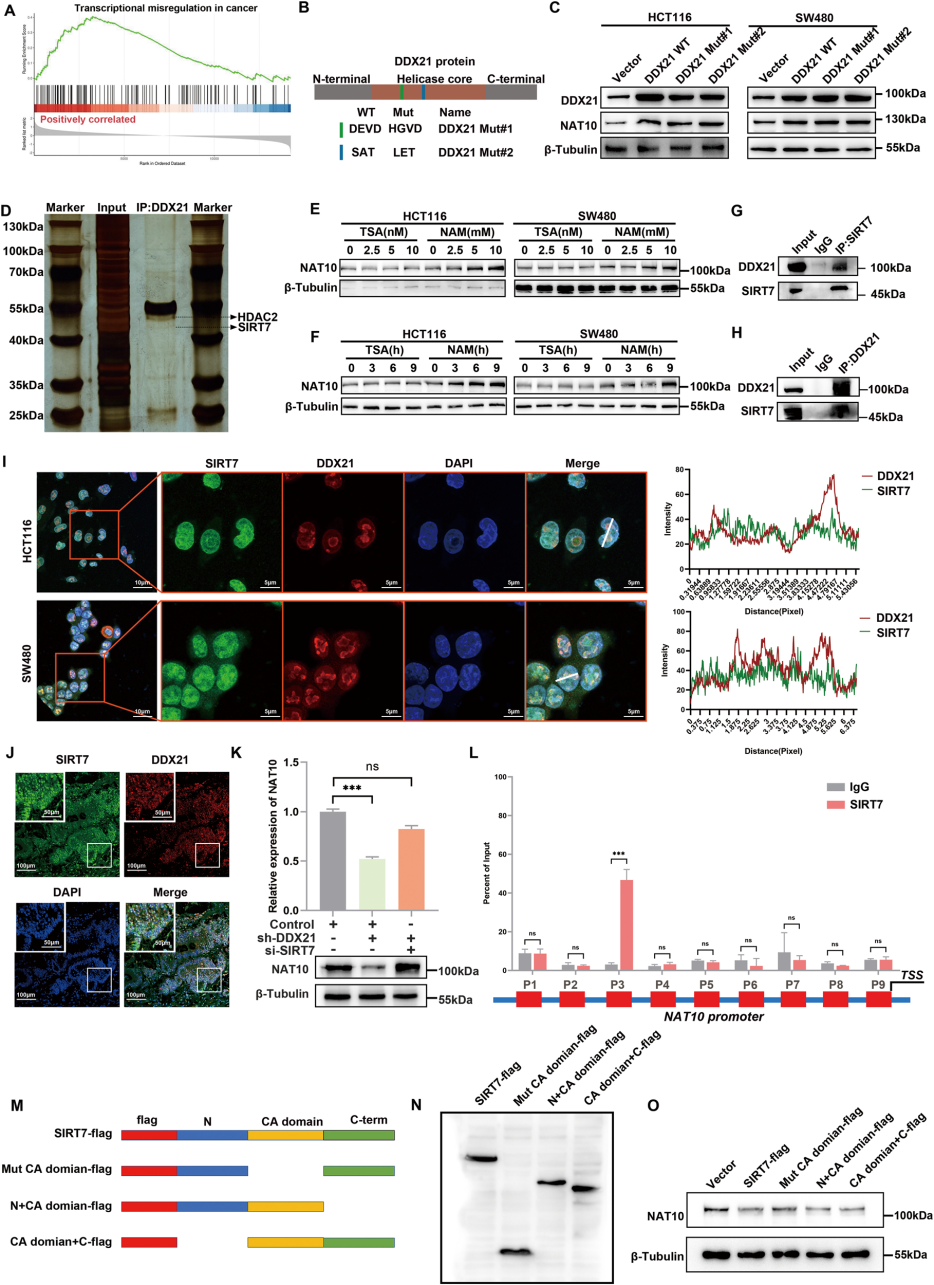
DDX21 competitively binds to the CA domain of Sirt7. **A** GSEA showed that DDX21 is related to “transcriptional misregulation in cancer”. **B**, **C** Based on DDX21 molecular structure, a series of mutants were designed to deactivate the helicase enzyme domain of DDX21. There was no significant difference in NAT10 protein expression among the DDX21 wild-type, helicase mutant DDX21#1 and ATP hydrolysis mutant DDX21#2 groups. **D** IP-MS assay was used to determine the interaction proteins of DDX21. **E**, **F** TSA and NAM were used to treat CRC cells. **G**, **H** IP and western blotting assays showed that DDX21 interacted with SIRT7. **I**, **J** IF assay showing the localization of DDX21 and SIRT7 in CRC cells (**I**) and tissues (**J**). DDX21 was labeled with CoraLite@594 (red), SIRT7 was labeled with CoraLite@488 (green), and nuclei were stained with DAPI (blue). Scale bars: 5 μm (**I**). Scale bars: 200 μm (**J**). **K** qRT‒PCR and western blotting were used to detect NAT10 mRNA and protein expression with DDX21 or SIRT7 silencing. **L** Based on the NAT10 promoter sequence, a series of primers were designed, Chip assay was used to detect the SIRT7 enrich site on NAT10 promoter. **M** A series of deletion mutants were constructed based on SIRT7 molecular function. **N** Western blotting showing the molecular weights of the SIRT7 mutants. **O** The mutants were transfected into CRC cells, and western blotting was used to determine the effect of the mutants on NAT10 protein expression. Data are presented as mean ± SD. *P < 0.05, **P < 0.01, ***P < 0.001, compared with the corresponding control group.

To investigate how DDX21 enhances NAT10 expression and thus regulates CRC metastasis and angiogenesis, we analyzed GESA of DDX21 RNA-seq. The results showed that DDX21 is related to “transcriptional misregulation in cancer” (Fig. 8A). Then, IF was performed to clarify the subcellular location of DDX21, and the results showed that DDX21 was located in the nucleus in CRC cells (Additional file 4: Fig. S6A). These findings evidenced that DDX21 transcriptionally activated NAT10 expression in CRC. Since DDX21 is an ATP-dependent RNA helicase, previous report confirmed that DDX21 promotes the release of positive transcription elongation factor b (P-TEFb) from the 7SK snRNP in a manner dependent on its helicase activity, thereby promoting the transcription of its target genes [28]. Then, we generated mutations in the DEAD-box (DEVD to HGVD) and SAT motifs (SAT to LET), which prevent ATP hydrolysis and helicase activity (Fig. 8B). Western blotting assays revealed that DDX21 enhances NAT10 expression independent of its RNA helicase activity (Fig. 8C). To further elucidate the mechanism by which DDX21 regulates NAT10 expression, Co-IP assay was performed in HCT116 cells. The enriched proteins were separated using western blotting for mass spectrometry (Co-IP/MS) (Fig. 8D). Moreover, HDAC2 and SIRT7 were identified as potential partners of DDX21. Thus, we hypothesized that DDX21 promoted NAT10 expression via HDAC2 or SIRT7. To further determine the mechanism by which DDX21 regulates NAT10 expression, we analyzed the expression level of NAT10 in CRC cells treated with trichostatin A (TSA), a broad-spectrum inhibitor of HDAC family deacetylases, and nicotinamide (NAM), an inhibitor of SIRT family deacetylases. The results showed that NAT10 expression is upregulated by NAM, which revealed that NAT10 expression might be inhibited by SIRT family deacetylases (Fig. 8E, F). Taken together, these results showed that DDX21 might regulate NAT10 expression via SIRT7.

Co-IP and western blotting assays were used to validate the physical interaction between DDX21 and SIRT7 (Fig. 8G, H). IF confirmed the colocalization of DDX21 and SIRT7 in CRC cells and tissues (Fig. 8J). Moreover, it was reported that SIRT7 inhibited gene expression through the deacetylation of histone H3 lysine 18 (H3K18) [29]. Western blotting and qRT–PCR assays revealed that the decrease in NAT10 levels caused by DDX21 knockdown could be reversed by SIRT7 knockdown in HCT116 cells (Fig. 8K). We hypothesized, based on our studies, that DDX21 might inhibit SIRT7-mediated deacetylation of H3K18 by competing with SIRT7 for binding to histone H3. Then, a series of truncation primers were designed based on the NAT10 promoter (P1, P2, P3, P4, P5, P6, P7, P8, and P9) (Fig. 8L). ChIP assays revealed that SIRT7 was enriched on the NAT10 promoter in P3 (Fig. 8L). Moreover, a ChIP assay was used to detect H3K18ac levels in the NAT10 promoter, which revealed that H3K18ac levels in the NAT10 promoter decreased with DDX21 knockdown and that H3K18ac levels were restored with SIRT7 knockdown (Additional file 4: Fig. S6B).

**Fig. 9:**
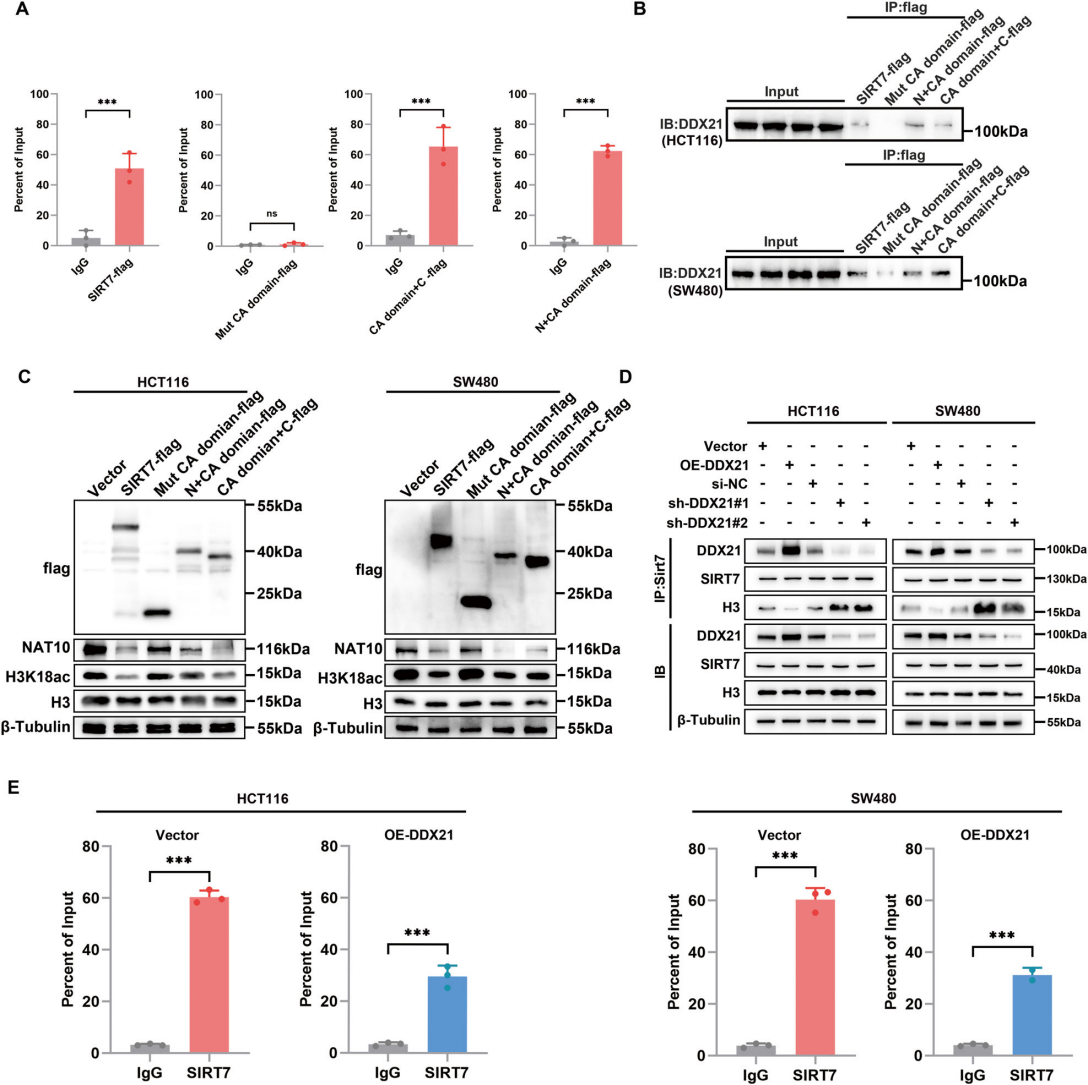
DDX21 suppressed NAT10 transcription by competitively binding to the CA domain of SIRT7. **A** ChIP assay confirmed that the CA domain of SIRT7 mediated the binding of the NAT10 promoter. **B** Mutants were transfected into CRC cells, and IP and western blotting confirmed that the CA domain of SIRT7 mediated the interaction with DDX21. **C** Vector and SIRT7 mutants were transfected, and the level of H3K18ac was detected. **D** Overexpression or silencing of DDX21 and the interaction of SIRT7 with histone H3 were detected. **E** ChIP assays were performed after DDX21 overexpression, and the binding efficiency was measured by qRT‒PCR. Data are presented as mean ± SD. *P < 0.05, **P < 0.01, ***P < 0.001, compared with the corresponding control group.

To clarify the molecular mechanism by which DDX21 regulates NAT10 via SIRT7, a series of deletion mutants were constructed of a series of Flag-tagged truncated SIRT7 proteins (Fig. 8M, N), and mutants within the catalytic domain (CA domain) of SIRT7 lacked of inhibition of NAT10 expression (Fig. 8O). Next, ChIP assays revealed that the SIRT7 CA domain deletion mutant failed to bind to the NAT10 promoter (Fig. 9A). On the other hand, a Co-IP assay was conducted with an anti-Flag antibody, and the results revealed that the CA domain of SIRT7 specifically interacts with DDX21 (Fig. 9B). Next, we performed western blotting experiments by reintroducing full-length or deletion mutants into CRC cells. Here, overexpression of Mut CA domain-FLAG reversed SIRT7-mediated NAT10 depletion and H3K18ac decrease (Fig. 9C). In addition, overexpression of DDX21 inhibited the binding of SIRT7 to histone H3; in contrast, DDX21 knockdown enhanced the interaction between SIRT7 and histone H3 (Fig. 9D). Furthermore, ChIP‒qPCR experiments demonstrated that the binding efficiency of SIRT7 to the NAT10 promoter was reduced after overexpression of DDX21 (Fig. 9E). These data indicated that DDX21 competitively binds to the CA domain of SIRT7, which mediates the deacetylation of the NAT10 promoter H3K18, thereby promoting NAT10 expression.

## Discussion

Accumulating evidence suggests that the DDX/DHX proteins are involved in CRC growth and metastasis and have emerged as promising targets for CRC therapy [30–32]. DDX21, one of the DDX/DHX proteins, shares a conserved “DEAD-box” sequence motif [33]. In this study, our comprehensive analysis of DDX/DHX proteins in the TCGA-CRC cohort led to the novel identification of DDX21 as a putative oncogene with extremely high expression levels in CRC, then we validated the upregulation of DDX21 in both CRC tissues and cell lines. Next, we found that high expression of DDX21 was positively associated with advanced clinicopathological stage and poor OS in CRC, suggesting that DDX21 might play an essential role in CRC progression. Moreover, animal models demonstrated that DDX21 enhanced CRC metastasis and growth, and decreased vascular invasion was observed in sh-DDX21-injected mouse tumors. Gain- and loss-of-function experiments identified that DDX21 promoted CRC metastasis and angiogenesis.

Furthermore, we provided evidence that the ac^4^C “writer”, NAT10, is a target gene of DDX21. NAT10 is a member of the GCN5-related N-acetyltransferase family. NAT10 has been described as a protein acetyltransferase that acetylates target proteins [22, 34], and subsequent studies revealed that NAT10 mediates RNA acetylation [35, 36]. Since NAT10 was confirmed to be an ac^4^C “writer”, it was demonstrated that NAT10 was upregulated in multiple tumors and enhanced mRNA stability and transcription [16, 19, 37]. However, the mechanism by which NAT10 is upregulated in tumors remains unknown. Here, we demonstrated that NAT10 expression was regulated by DDX21. This is the first report demonstrating the regulatory relationship between DDX21 and NAT10 and the first study to reveal the upstream mechanism of NAT10 in CRC.

Ac^4^C was initially detected on tRNA and rRNA [38, 39]. In recent years, it was also found to be widely present in human and yeast mRNAs [36, 40]. Increasing evidence indicates that ac^4^C modification is associated with tumor development and progression [16, 19, 36, 37]. However, the biological functions and regulatory impact of ac^4^C modification in CRC remain largely unknown. In the present study, we demonstrated that both the ac^4^C modification in RNA and its writer NAT10 are overexpressed in CRC. Our RNA-seq and acRIP-seq high-throughput analyses and subsequent validation and functional studies suggested that the oncogenes ATAD2, SOX4, and SNX5 were targets of NAT10. NAT10-mediated ac^4^C modification of ATAD2, SOX4, and SNX5 mRNAs at the CDS and 3’UTR region, increasing the mRNAs stability, thereby leading to the upregulation of oncogenes and inducing metastasis and angiogenesis in CRC. Moreover, acRIP-seq analysis confirmed that ac^4^C modification mainly enriched in the 3’ UTR and CDS regions. Knockdown of NAT10, the ac^4^C writer, mainly influenced the 3’ UTR and CDS enrichment of ac^4^C, but the 5’ UTR region seemed less affected. At the same time, it was found that NAT10 silencing failed to significantly weaken ac^4^C modification in mRNAs, confirming the presence of other “writers” of ac^4^C modification. It will be interesting to explore the possible ac^4^C writers, which will expand our understanding of the regulatory network of ac^4^C acetylation.

As an NAD^+^-dependent HDAC, SIRT7 controls a wide range of cellular functions through the deacetylation of its substrates. For example, SIRT7 targets PAF53, which modulates ribosomal DNA occupancy of polymerase I (Pol I) and transcription activation [41]. Recent studies have also revealed a desuccinylase function of SIRT7 [42]. Several studies have also confirmed that SIRT7 drives histone deacetylation [43–46], and mediates the transcriptional repression of several tumor suppressor genes [45]. In CRC, Yu et al. revealed that SIRT7 enhanced MAPK pathway activity and promoted CRC metastasis [47]. In the present study, we demonstrated that DDX21 blocked the SIRT7-mediated decrease in H3K18ac in the NAT10 promoter, enhancing the transcription of NAT10. This result indicated that SIRT7 might inhibit CRC metastasis and angiogenesis. Overall, the function of SIRT7 in cancer is determined by different target proteins, and the role of SIRT7 in CRC needs further exploration.

Overall, these results indicated that DDX21 was significantly upregulated in CRC. The CA domain of SIRT7 mediates its interaction with DDX21 and histone H3, while DDX21 competitive binding the CA domain of SIRT7, the histone H3 deacetylase, to block the SIRT7-mediated transcriptional inhibition of NAT10. NAT10 remodeled the ac^4^C modification of the mRNAs of the oncogenic genes ATAD2, SOX4, and SNX5, which have been confirmed to play important roles in tumor metastasis and angiogenesis. In summary, our findings indicated that the DDX21-induced ac^4^C modification axis is an important regulator of CRC metastasis and angiogenesis; hence, blocking the DDX21/NAT10 axis is a promising strategy for CRC treatment.

## Supplementary information

SUPPLEMENTAL MATERIAL

## Data Availability

The datasets used and/or analyzed during the current study are available from the corresponding author upon reasonable request.
