# Supplementary material for: Competitive binding between DDX21 and SIRT7 enhances NAT10-mediated ac4C modification to promote colorectal cancer metastasis and angiogenesis– DDX21 promotes colorectal cancer metastasis
Source: Cell Death Dis. 2025 Apr 29;16(1):353. doi: 10.1038/s41419-025-07656-3 (PMC12041575; doi:10.1038/s41419-025-07656-3)

Additional file 1

Table S1, Relationship between DDX21 expression and clinicopathological features in TMAs specimens.

Variables	Cases	DDX21 expression (n=73)		p^a
		Low (n=37)	High (n=36)	
Age				0.6771
< 60	24	13	11	
⩾60	49	24	25	
Gender				0.9130
Female	39	20	19	
Male	34	17	17	
Tumor diameter (cm)				**0.0112**
⩽5cm	29	20	9	
> 5cm	44	17	27	
Depth of invasion				**< 0.0001**
T1/T2	41	30	11	
T3/T4	32	7	25	
Lymph node metastasis				**0.0064**
N0	44	28	16	
N1/N2	29	9	20	
Distant metastasis				**0.0027**
M0	54	33	21	
M1	19	4	15	
TNM stage				**0.0140**
I/II	37	24	13	
III/IV	36	13	23	
Differentiation				0.9130
Middle/High	34	17	17	
Poor	39	20	19	

p-value [a] was measured by Pearson's Chi-Squared test.

Additional file 2

Table S2, Relationship between DDX21 expression and clinicopathological features in fresh frozen tissues.

**Variables**	**Cases**	**DDX21 expression (n=52)**		p^a
		**Low (n=26)**	**High (n=26)**	
Age				0.397
＜60	21	9	12	
⩾60	31	17	14	
Gender				0.578
Female	28	15	13	
Male	24	11	13	
Tumor diameter (cm)				**0.012**
⩽5cm	23	16	7	
＞5cm	29	10	19	
Depth of invasion				**0.002**
T1/T2	23	17	6	
T3/T4	29	9	20	
Lymph node metastasis				**<0.001**
N0	20	18	2	
N1/N2	32	8	24	
Distant metastasis				**0.042**
M0	45	25	20	
M1	7	1	6	
TNM stage				**<0.001**
I/II	22	17	5	
III/IV	30	9	21	
Differentiation				0.780
Middle/High	23	12	11	
Poor	29	14	15	

p-value [a] was measured by Pearson's Chi-Squared test.

Additional file 3

Table S3, siRNA sequences, shRNA sequences and primer sequences and mutants were listed.

SiRNAs/shRNAs

sh-DDX21#1 5’-GGAAUUAAGUUCAAACGAAUA-3’

sh-DDX21#2 5’-GCAUGUAUCUGCCUAUACUUU-3’

si-NAT10#1 5’-CCGAAUCCGGAUUCUCAUU-3’

si-NAT10#2 5’-GGCCAAAGCUGUCUUGAAA-3’

si-SIRT7 5’-AGCCAUUUGUCCUUGAGGAA-3’

Primers

DDX21 Forward primer: 5’- TCATCAAGGACGCACTATCATCT -3’

DDX21 Reverse primer: 5’- CCTTTCAGGGTGATTTCCCTTT -3’

SIRT7 Forward primer: 5’- ATGAGCAGAAGCTGGTGC -3’

SIRT7 Reverse primer: 5’- CTGTCTGGTGTCTGTGGA -3’

NAT10 Forward primer: 5’- CATACGCTAGGGGCAGTCAG -3’

NAT10 Reverse primer: 5’- TCTTTGCCGCTCAGCTACTC -3’

SOX4 Forward primer: 5’- CAGCAAACCAACAATGCCGA -3’

SOX4 Reverse primer: 5’- GATCTGCGACCACACCATGA -3’

SNX5 Forward primer: 5’- GCGGTTCCCGAGTTGCT -3’

SNX5 Reverse primer: 5’- GCAGTGTGGTCTTTGTGTGC -3’

ATAD2 Forward primer: 5’- GAGCTGGATCTCTCTCCGGT -3’

ATAD2 Reverse primer: 5’- TAACTGATGACCCATCGCCC -3’

NAT10 ChIP 1 Forward primers: 5’- TCAGCTATTTGAGAGGCTGAGG -3’

NAT10 ChIP 1 Reverse primers: 5’- GGTAACTTTATTAAGTGAGCCACCAAC -3’

NAT10 ChIP 2 Forward primers: 5’- GTTACCATTTAAAGAACATTCAATGCAA -3’

NAT10 ChIP 2 Reverse primers: 5’- AGAGATGAAGTCTGACATCCAAGAC -3’

NAT10 ChIP 3 Forward primers: 5’- ATCTCTAGCTGTGTTCTTCCCTG -3’

NAT10 ChIP 3 Reverse primers: 5’- GCACCCAAAACATTTGCTCTGA -3’

NAT10 ChIP 4 Forward primers: 5’- TGGGTGCCATCTTCAATGTACTT -3’

NAT10 ChIP 4 Reverse primers: 5’- AGGGAAATGAGAAAATCTGCTTTGC -3’

NAT10 ChIP 5 Forward primers: 5’- GTCCCCCACAAACTCCAGTG -3’

NAT10 ChIP 5 Reverse primers: 5’- CGAACACTGAAGGCCTCTGAC -3’

NAT10 ChIP 6 Forward primers: 5’- GTGTTCGTTTCTAAAATAGTATCCTTAGT -3’

NAT10 ChIP 6 Reverse primers: 5’- CAGCAGCACTACCCATTTAATCC -3

NAT10 ChIP 7 Forward primers: 5’- GTGCTGCTGATGACATTAAAAGTG -3’

NAT10 ChIP 7 Reverse primers: 5’- CATTAGGCCTGTATATGGTCGTC -3

NAT10 ChIP 8 Forward primers: 5’- GGAGCCTGCAAAGTACAGC -3’

NAT10 ChIP 8 Reverse primers: 5’- CCGCACCCCAGGGC -3

NAT10 ChIP 9 Forward primers: 5’- ACATCTATTTTGCCCTTCTTAGCC -3’

NAT10 ChIP 9 Reverse primers: 5’- GTGCGCAAGCGCATTAAAG -3’

NAT10 RIP Forward primer: 5’- CAGGCTTACTACGTGACCCG -3’

NAT10 RIP Reverse primer: 5’- TCCGGATTCGGTTATCCACC -3’

18S Forward primers: 5’- GTAACCCGTTGAACCCCATT -3’

18S Reverse primers: 5’- CCATCCAATCGGTAGTAGCG -3’

GAPDH Forward primers: 5’- GAAGGTGAAGGTCGGAGTC -3’

GAPDH Reverse primers: 5’- GAAGATGGTGATGGGATTTC -3’

Mutants

Sirt7 full-length

MAAGGLSRSERKAAERVRRLREEQQRERLRQVSRILRKAAAERSAEEGRLLA

ESADLVTELQGRSRRREGLKRRQEEVCDDPEELRGKVRELASAVRNAKYLV

VYTGAGISTAASIPDYRGPNGVWTLLQKGRSVSAADLSEAEPTLTHMSITRLH

EQKLVQHVVSQNCDGLHLRSGLPRTAISELHGNMYIEVCTSCVPNREYVRVF

DVTERTALHRHQTGRTCHKCGTQLRDTIVHFGERGTLGQPLNWEAATEAAS

RADTILCLGSSLKVLKKYPRLWCMTKPPSRRPKLYIVNLQWTPKDDWAALK

LHGKCDDVMRLLMAELGLEIPAYSRWQDPIFSLATPLRAGEEGSHSRKSLCRS

REEAPPGDRGAPLSSAPILGGWFGRGCTKRTKRKKVT

Sirt7 MUT CA domain

MAAGGLSRSERKAAERVRRLREEQQRERLRQVSRILRKAAAERSAEEGRLLA

ESADLVTELQGRSRRREGLKRRQEEVCDDPEELRGKVRAYSRWQDPIFSLAT

PLRAGEEGSHSRKSLCRSREEAPPGDRGAPLSSAPILGGWFGRGCTKRTKRKK

VT

Sirt7 N+CA domain

MAAGGLSRSERKAAERVRRLREEQQRERLRQVSRILRKAAAERSAEEGRLLA

ESADLVTELQGRSRRREGLKRRQEEVCDDPEELRGKVRELASAVRNAKYLV

VYTGAGISTAASIPDYRGPNGVWTLLQKGRSVSAADLSEAEPTLTHMSITRLH

EQKLVQHVVSQNCDGLHLRSGLPRTAISELHGNMYIEVCTSCVPNREYVRVF

DVTERTALHRHQTGRTCHKCGTQLRDTIVHFGERGTLGQPLNWEAATEAAS

RADTILCLGSSLKVLKKYPRLWCMTKPPSRRPKLYIVNLQWTPKDDWAALK

LHGKCDDVMRLLMAELGLEIP

Sirt7 CA domain+ C

ELASAVRNAKYLVVYTGAGISTAASIPDYRGPNGVWTLLQKGRSVSAADLSE

AEPTLTHMSITRLHEQKLVQHVVSQNCDGLHLRSGLPRTAISELHGNMYIEVC

TSCVPNREYVRVFDVTERTALHRHQTGRTCHKCGTQLRDTIVHFGERGTLGQ

PLNWEAATEAASRADTILCLGSSLKVLKKYPRLWCMTKPPSRRPKLYIVNLQ

WTPKDDWAALKLHGKCDDVMRLLMAELGLEIPAYSRWQDPIFSLATPLRAGE

EGSHSRKSLCRSREEAPPGDRGAPLSSAPILGGWFGRGCTKRTKRKKVT

Additional file 4

Supplement Figures

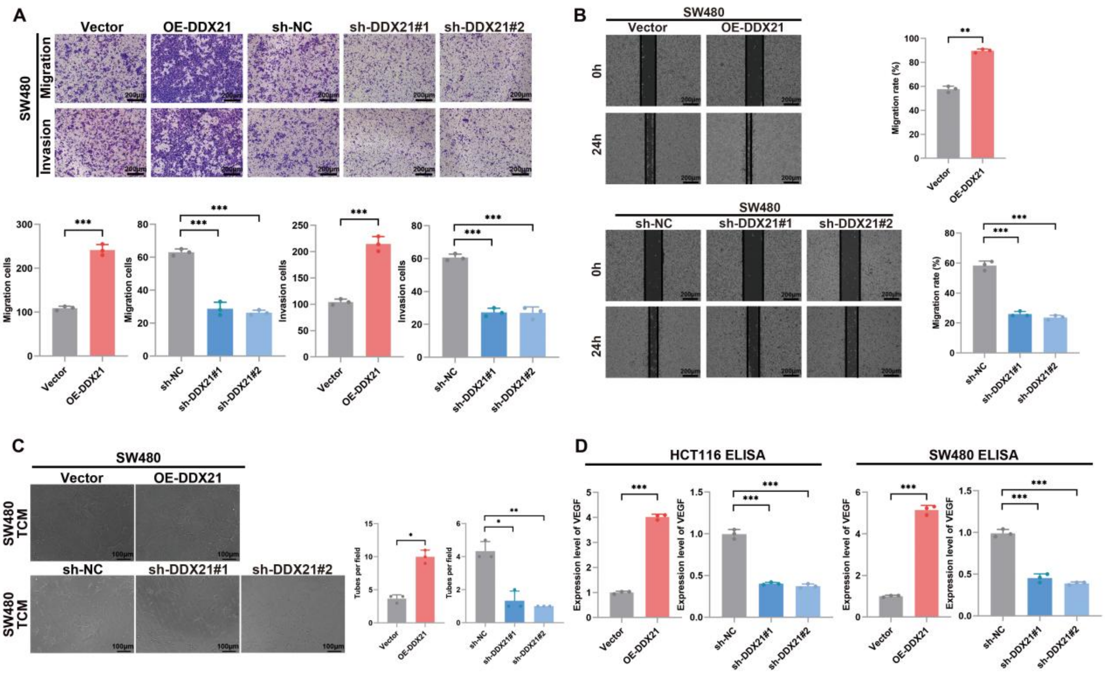

Fig. S1 DDX21 promoted CRC cells metastasis and angiogenesis.

A The effects of DDX21 on cell invasion and migration were analyzed by Transwell assay. Scale bars: 200 μm. **B** The effects of different expression levels of DDX21 on cell migration were analyzed by wound healing assay. Scale bars: 200 μm. **C** Tube formation assay was used to analyze the effects of DDX21 on angiogenesis of CRC cells. Scale bars: 100 μm. **D** ELISA assay showed that DDX21 overexpression promoted VEGF expression while DDX21 silencing significantly inhibited VEGF expression. Data are presented as mean ± SD. *P < 0.05, **P < 0.01, ***P < 0.001, compared with the corresponding control group.

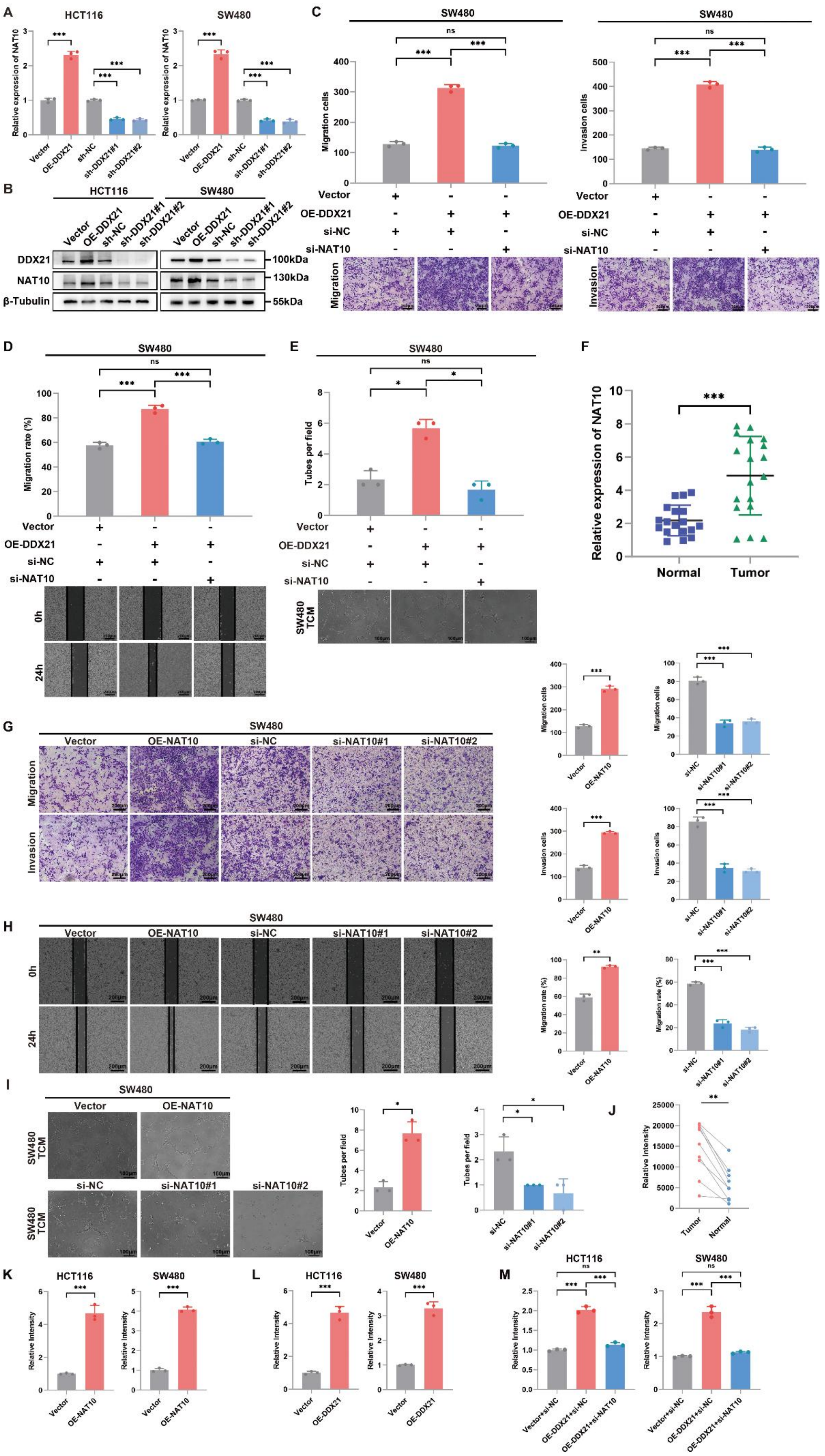
A
HCT116
SW480
Relative expression of NAT10
Vector
OE-DDX21
sh-NC
sh-DDX21#1
sh-DDX21#2
B
DDX21
NAT10
β-Tubulin
100kDa
130kDa
55kDa
C
Migration cells
Invasion cells
si-NC
si-NAT10
Migration
Invasion
D
Migration rate (%)
0h
24h
E
Tubes per field
SW480 TCM
F
Relative expression of NAT10
Normal
Tumor
G
OE-NAT10
si-NAT10#1
si-NAT10#2
H
I
J
Relative Intensity
K
L
M
Vector+si-NC
OE-DDX21+si-NC
OE-DDX21+si-NAT10
ns

Fig. S2 NAT10 was the target of DDX21 and promotes metastasis and angiogenesis of CRC.

A-B qRT-PCR and western blot assays were used to determine the levels of NAT10 in DDX21-overexpressing and DDX21-silenced cells. **C** Knockdown of NAT10 was performed in DDX21-overexpressing CRC cells, and the invasion and migration capacity of the cells was analyzed by Transwell assay. Scale bars: 200 μm. **D** Knockdown of NAT10 was performed in DDX21-overexpressing CRC cells, and the ability of cell migration was analyzed by wound healing assay. Scale bars: 200 μm. **E** Knockdown of NAT10 was performed in DDX21-overexpressing CRC cells, and the ability of angiogenesis was analyzed by tube formation assay. Scale bars: 100 μm. **F** NAT10 mRNA expression level was determine using qRT-PCR. **G** The effects of NAT10 on cell invasion and migration were analyzed by Transwell assay. Scale bars: 200 μm. **H** The effects of different expression levels of NAT10 on cell migration were analyzed by wound healing assay. Scale bars: 200 μm. **I** Tube formation assay was used to analyze the effects of NAT10 on angiogenesis of CRC cells. Scale bars: 100 μm. **J** Dot blot assays were performed using RNA extracted from tumors and normal tissues, and the intensity of the blotting signals was quantified through gray-scale analysis and statistical analysis. **K-M** The gray-scale analysis of the relevant dot blot assays was normalized and then subjected to statistical analysis. Data are presented as mean ± SD. *$P < 0.05$, **$P < 0.01$, ***$P < 0.001$, compared with the corresponding control group.

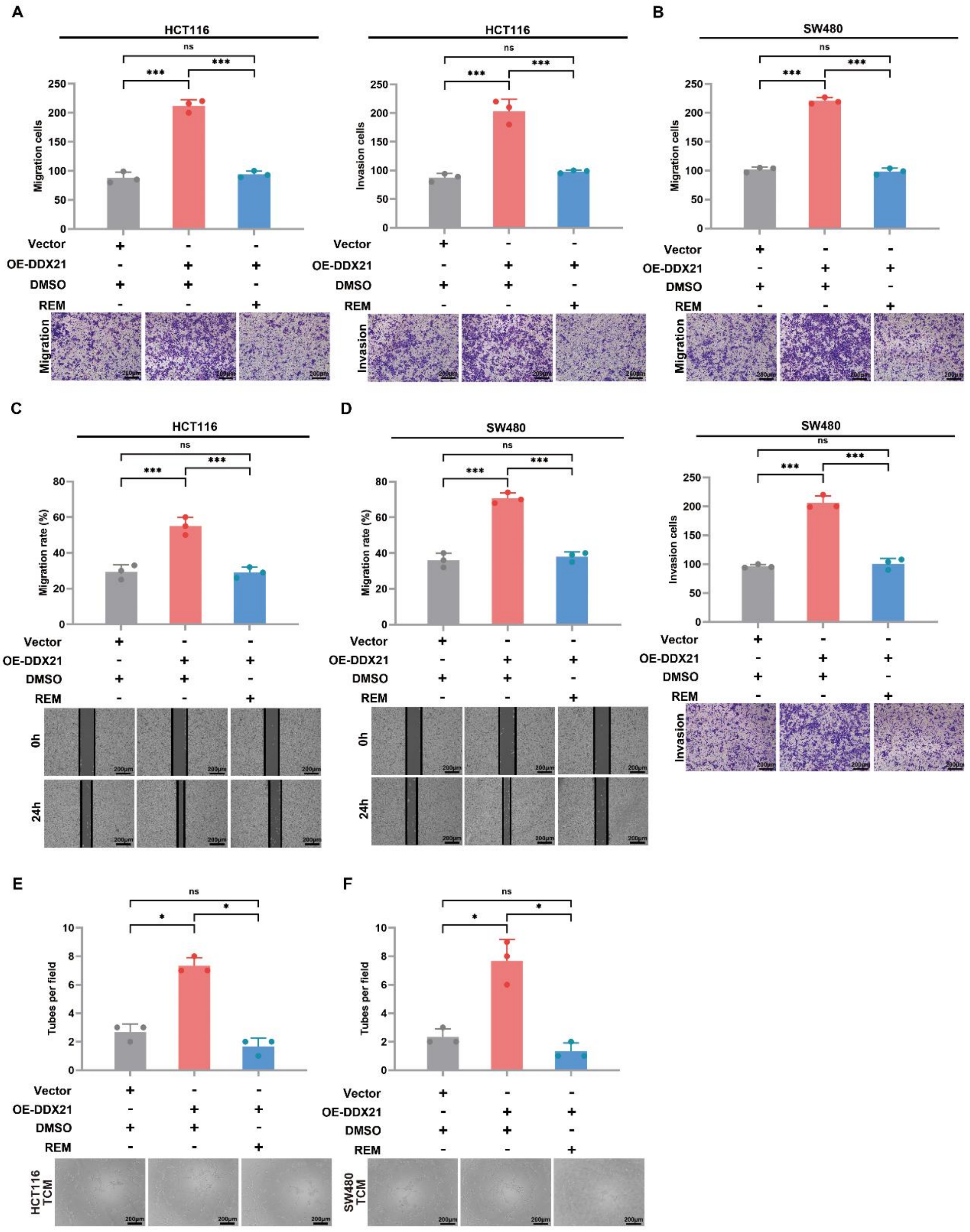

Fig. S3 DDX21 promoted CRC metastasis and angiogenesis through NAT10.

A-B REM treated DDX21-overexpressing CRC cells, Transwell assay was used to detect CRC cells migration and invasion ability. Scale bars: 200 μm. **C-D** REM treated DDX21-overexpressing CRC cells, wound healing assay was used to detect CRC cells migration ability. Scale bars: 200 μm. **E-F** REM treated DDX21-overexpressing CRC

cells, tube formation assay was used to detect CRC cells angiogenesis ability. Scale bars: 100 μm. Data are presented as mean ± SD. *P < 0.05, **P < 0.01, ***P < 0.001, compared with the corresponding control group.

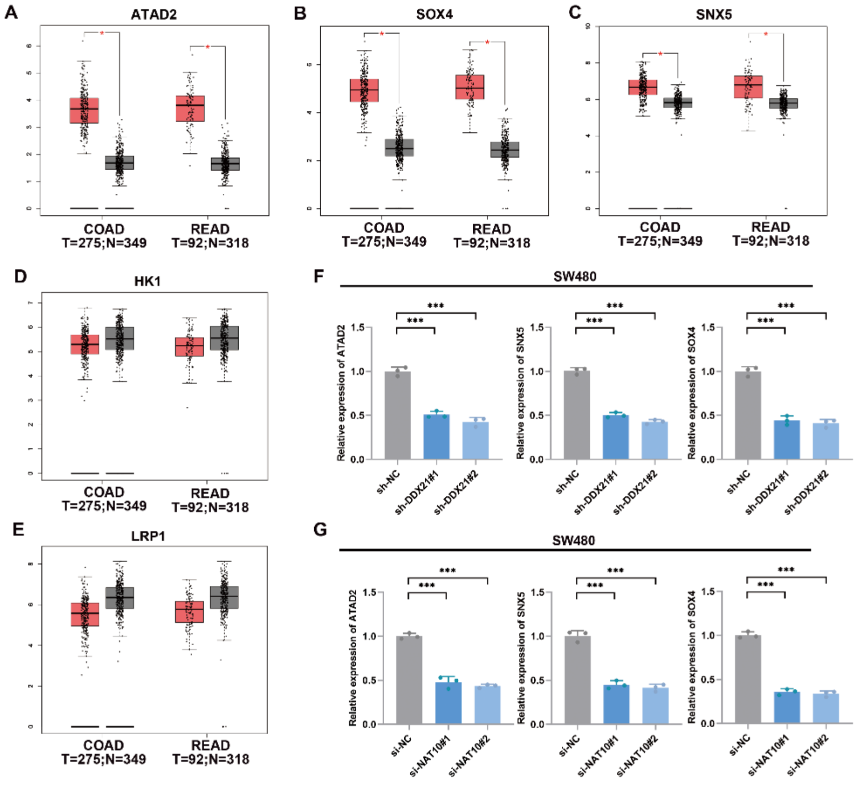

Fig. S4 ATAD2, SOX4 and SNX5 were upregulated in CRC while HK1 and LRP1 were no significant difference in CRC and normal tissues.

A-E DDX21 expression level in COAD (T=275; N=349) and READ (T=92; N=318) were identified in GEPIA database, *P < 0.05, **P < 0.01 and ***P < 0.001. **F** DDX21 knockdown inhibited mRNAs expression of ATAD2, SOX4 and SNX5 in SW480 cells. **G** NAT10 knockdown inhibited mRNAs expression of ATAD2, SOX4 and SNX5 in SW480 cells. Data are presented as mean ± SD. *P < 0.05, **P < 0.01, ***P < 0.001, compared with the corresponding control group.

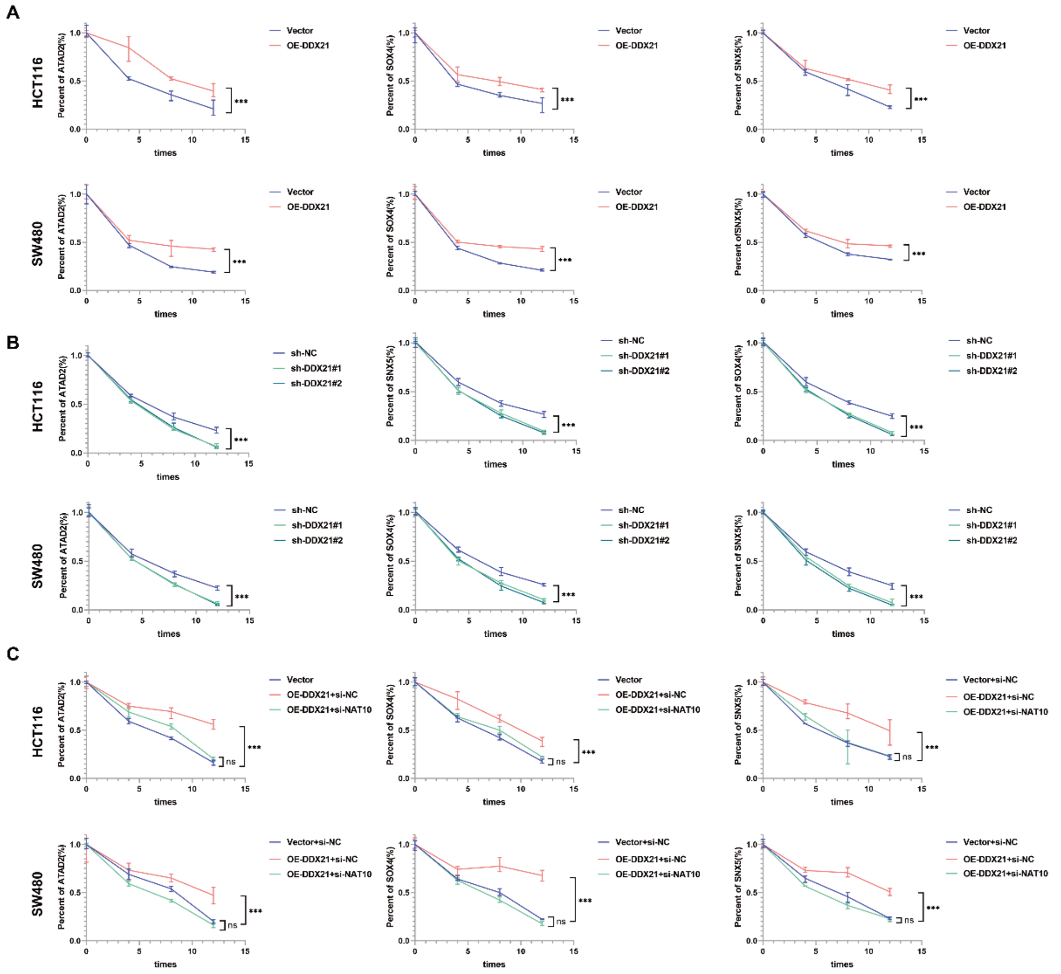

Fig. S5 DDX21 promoted ATAD2, SOX4 and SNX5 mRNAs stability via NAT10-mediated ac^{4}C modification.

A, B The mRNA stability assays were adopted to detect the relative level of mRNAs after treatment with DDX21-overexpressing or DDX21-silenced CRC cells. **C** The mRNA stability assays were used to determine the ATAD2, SOX4 and SNX5 mRNAs stability of DDX21-overexpressing CRC cells transfected with NAT10 siRNA. Data are presented as mean ± SD. **P* < 0.05, ***P* < 0.01, ****P* < 0.001, compared with the corresponding control group.

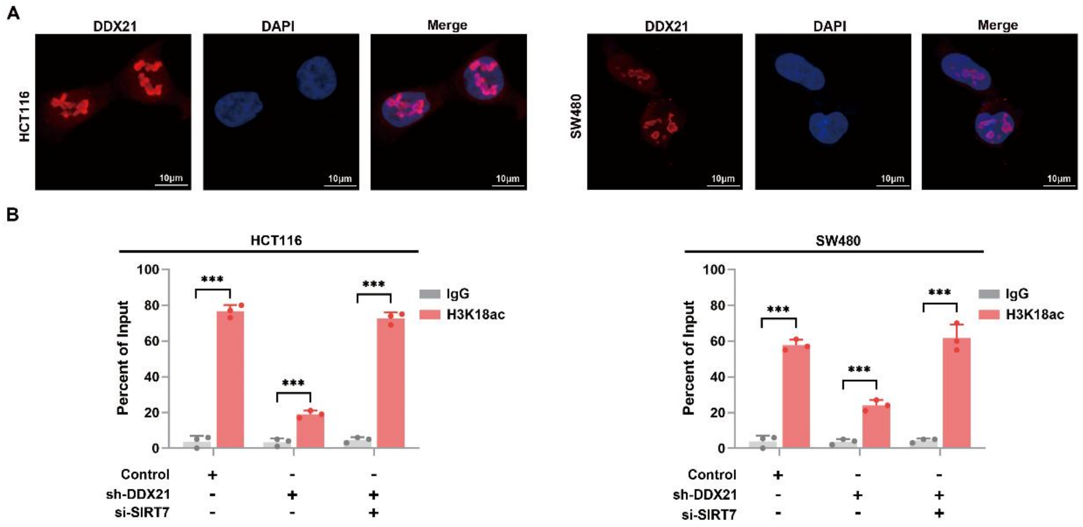

Fig. S6 DDX21 inhibited SIRT7-mediated H3K18ac of NAT10 promoter.

A Representative image for IF staining of DDX21 in CRC cells. The DDX21 was labeled with CoraLite@594 (red); nuclei were stained with DAPI (blue). Scale bars: 10 µm. **B, C** DDX21 silencing inhibited the level of H3K18ac of NAT10 promoter, SIRT7 silencing promoted NAT10 promoter H3K18ac. ChIP assay confirmed that the H3K18ac level reversed in DDX21-silenced CRC cells transfected with SIRT7 siRNA. Data are presented as mean ± SD. *$P < 0.05$, **$P < 0.01$, ***$P < 0.001$, compared with the corresponding control group.

Additional file 5

Original Western blot assays

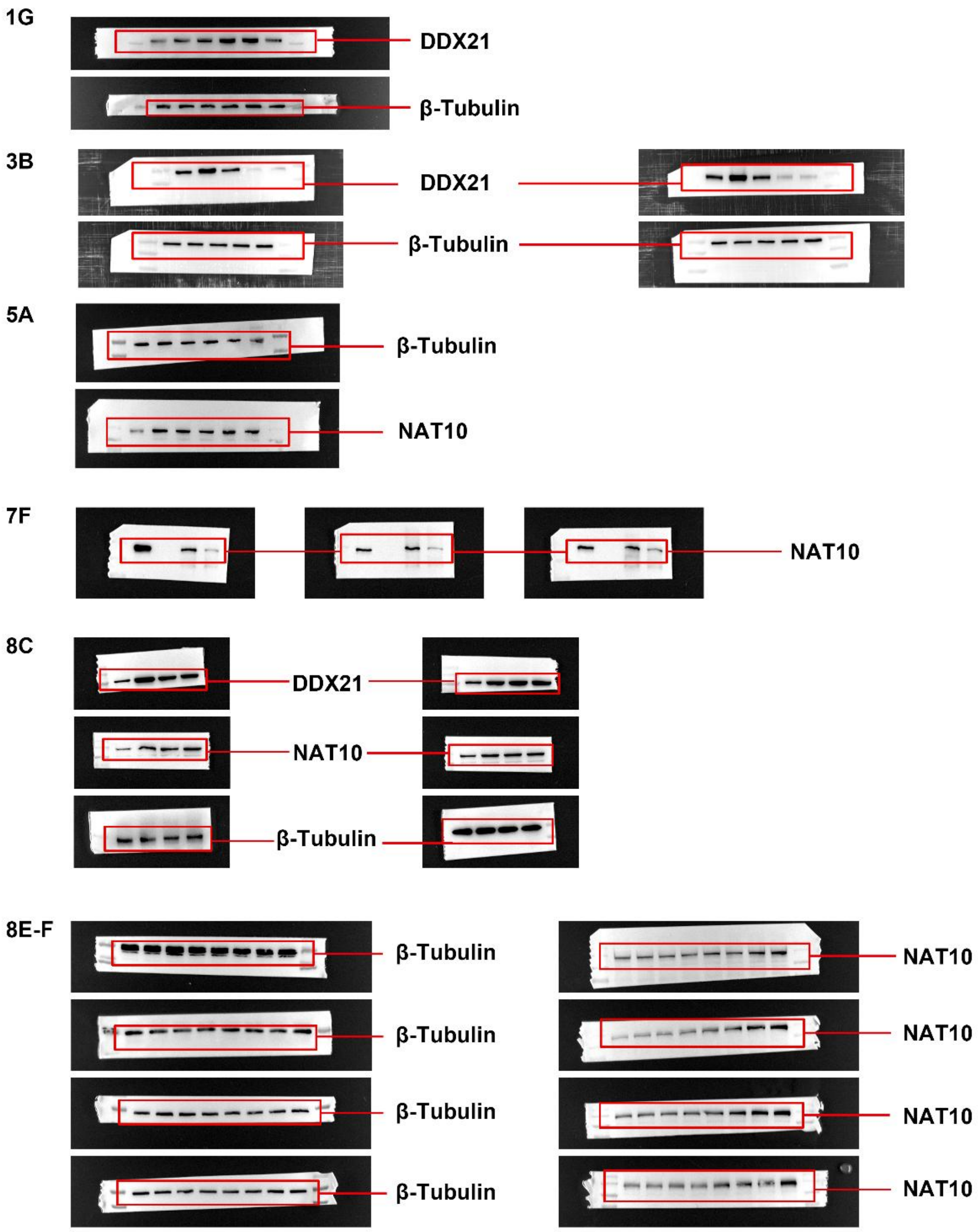

8G-H

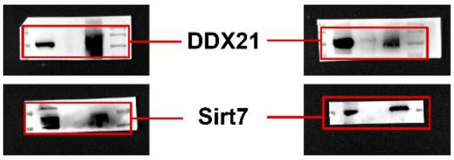

8K

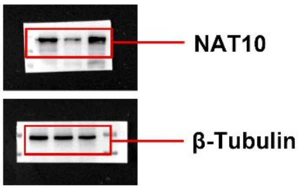

8N

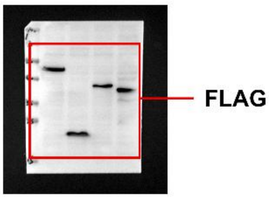

8O

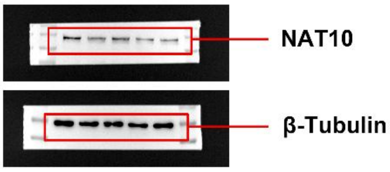

9B

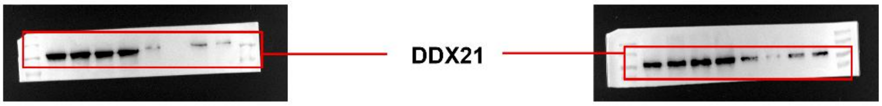

9C

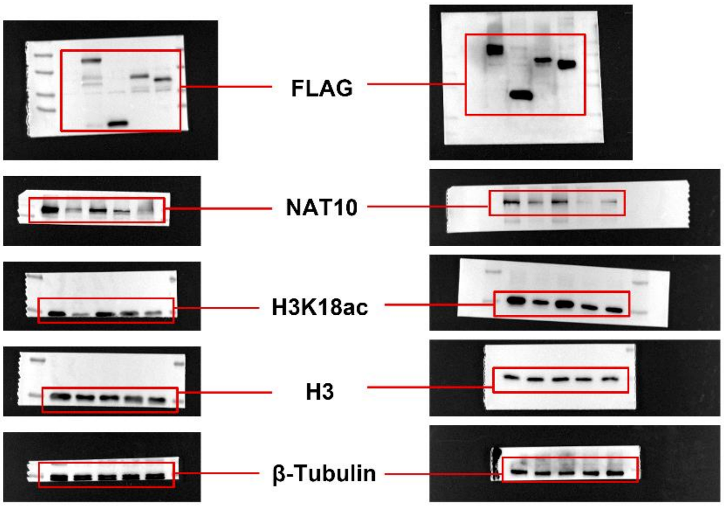

FLAG
NAT10
H3K18ac
H3
β-Tubulin

9D

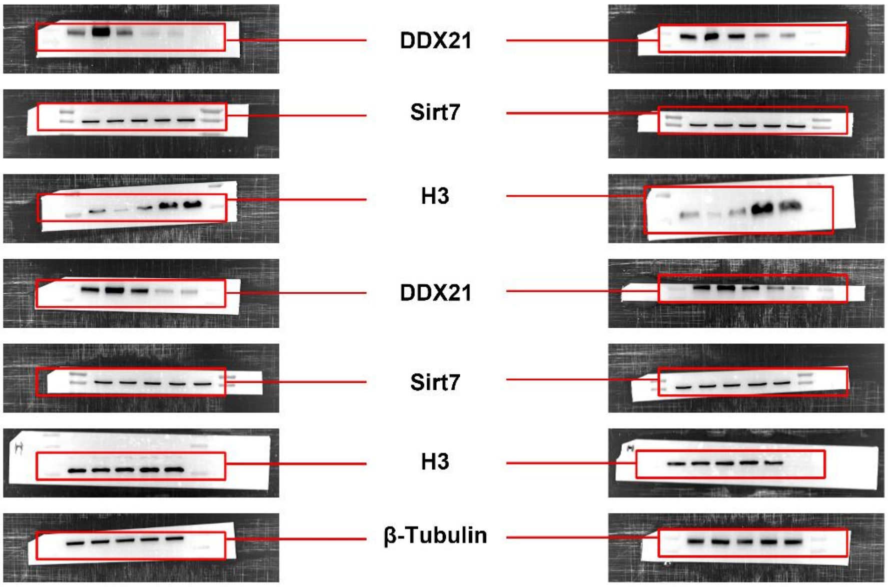

DDX21
Sirt7
H3
DDX21
Sirt7
H3
β-Tubulin

S2B

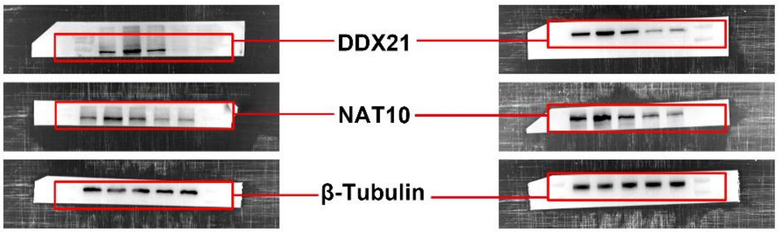

Supplement: Supplementary file 1 — SUPPLEMENTAL MATERIAL [file 41419_2025_7656_MOESM1_ESM.pdf]
